# Developing a frame of reference for fisheries management and conservation interventions

**DOI:** 10.1016/j.fishres.2018.08.010

**Published:** 2018-12

**Authors:** Annabelle Jade Bladon, Essam Yassin Mohammed, Liaquat Ali, E.J. Milner-Gulland

**Affiliations:** a Imperial College London, Silwood Park Campus, Buckhurst Road, Ascot, Berkshire, UK; b International Institute for Environment and Development, London, UK; c Bangladesh Centre for Advanced Studies, Dhaka, Bangladesh; d Department of Zoology, University of Oxford, Oxford, UK

**Keywords:** Counterfactual, Uncertainty, Baseline, Hilsa, Bangladesh

## Abstract

Effective implementation of management interventions is often limited by uncertainty, particularly in small-scale and developing-world fisheries. An effective intervention must have a measurable benefit, and evaluation of this benefit requires an understanding of the historical and socio-ecological context in which the intervention takes place. This context or ‘frame of reference’ should include the baseline status of the species of interest, as well as the most likely counterfactual (a projected scenario indicating what would have occurred in the absence of the intervention), given recent trends. Although counterfactuals are difficult to estimate and so are not widely specified in practice, an informative frame of reference can be developed even in data-poor circumstances. We demonstrate this using a case study of the Bangladesh hilsa (*Tenualosa ilisha*) fishery. We combine qualitative and some quantitative analyses of secondary datasets to explore ecological trends in the hilsa fishery, as well as patterns of social, economic, institutional, and physical change relevant to its management over the last ∼50 years. We compile all available information on the key parameters that determine hilsa abundance and distribution (movement, reproduction, growth, and mortality), as well as all available information on stock status. This information is used to produce a baseline and qualitative counterfactual which can be used to guide decision-making in this complex, data-poor fishery. A frame of reference provides a systematic way to break down potential drivers of change in a fishery, including their interactions, reducing the potential for unexpected management outcomes. Critical evaluation of contradictions and commonalities between a set of potential counterfactuals, as well as the reliability of sources, allows the identification of key areas of uncertainty and information needs. These can then be incorporated into fisheries management planning.

## Highlights

• A frame of reference provides necessary context for conservation interventions.
• This approach has never been used in the fisheries literature.
• We develop a quantitative frame of reference for the Bangladesh hilsa fishery.
• This is shown to support decision-making, even in data-poor circumstances.
• The frame of reference highlights uncertainties and information needs.

## 1 Introduction

An effective conservation or management intervention should have a measurable benefit, and thus requires the specification of an appropriate frame of reference against which it can be evaluated (Bull et al., 2014; Maron et al., 2013). A robust frame of reference should include a baseline that expresses conditions at a fixed point in time (whether a current or past reference state), and one or more counterfactuals (dynamic baselines or scenarios that use background rates of change to estimate potential states of a system in the absence of an intervention; Bull et al., 2016). This baseline and counterfactual should capture ongoing trends in the ecological status of the intervention target (in fisheries management, this will usually be a specific species of interest); as well as the institutional, social, economic, and physical factors driving these trends; and the potential interactions and feedbacks between these factors (Bull et al., 2015; Ferraro and Pattanayak, 2006; Nicholson et al., 2009). Ideally, the development and evaluation of any intervention should consider the historical context within which it operates, using major events and system shifts to understand the main factors driving the dynamics of the social-ecological system (Bull et al., 2015; Pooley, 2013).

Prior development of a counterfactual enables the rigorous measurement and attribution of impact, i.e. the difference between the outcome of the intervention and the estimated outcome in the absence of the intervention (Bull, 2014; Bull et al., 2015; Pattanayak et al., 2010). However, counterfactuals are rarely developed early in the intervention design process and, when they are, they often contain incorrect or vague assumptions (Gurney et al., 2015, 2014; Maron et al., 2013). A common reason for this is lack of data; counterfactuals are subject to numerous sources of uncertainty and it can be challenging to develop and validate projected trends when knowledge is poor (Bull et al., 2015, 2014). In fisheries management, uncertainties often limit the ability of policymakers to project trends and predict the effects of management interventions (Davies et al., 2015). These challenges are particularly pronounced in small-scale and developing-world fisheries, where data limitations mean even fixed baselines can be difficult to estimate (Carruthers et al., 2014). Useful counterfactuals can nevertheless be developed in these circumstances, as long as assumptions and limitations are acknowledged; and the process of developing them can highlight key areas of uncertainty which might hinder the development of effective interventions and limit evaluations (Bull et al., 2015, 2014).

Bull et al. (2015) conceptualized and demonstrated a structure for developing a frame of reference in the context of terrestrial biodiversity offsetting. This paper uses the hilsa (*Tenualosa ilisha*) fishery in Bangladesh as a case study to demonstrate the wider utility of this approach in conservation, and its potential value for fisheries management, even when data are limited. The hilsa fishery is currently managed through a combination of regulatory measures, as well as a fisher rehabilitation programme that aims to incentivize compliance with these regulations (Section 3.5.1 and mmc2 in Supplementary material), but since no counterfactuals were developed before the introduction of these interventions, attempts to evaluate impact have lacked rigor (Bladon et al., 2016). The frame of reference developed in this paper combines qualitative and some quantitative analyses of secondary datasets and literature in a qualitative way to explore: a) patterns of social, economic, institutional and physical change relevant to the management of hilsa in Bangladesh; and b) ecological trends in the hilsa fishery. Two plausible qualitative counterfactuals are put forward, which could be used to evaluate potential future hilsa management and conservation interventions.

## 2 Methods

This frame of reference is structured following the framework of Bull et al. (2015) who demonstrated the approach in the context of biodiversity offsets for the residual ecological impacts of oil and gas extraction in Uzbekistan. We adapted the framework slightly to fit the case study, e.g. we focused on a species target, not a habitat target.

### 2.1 Brief recent history

**Fig. 1:**
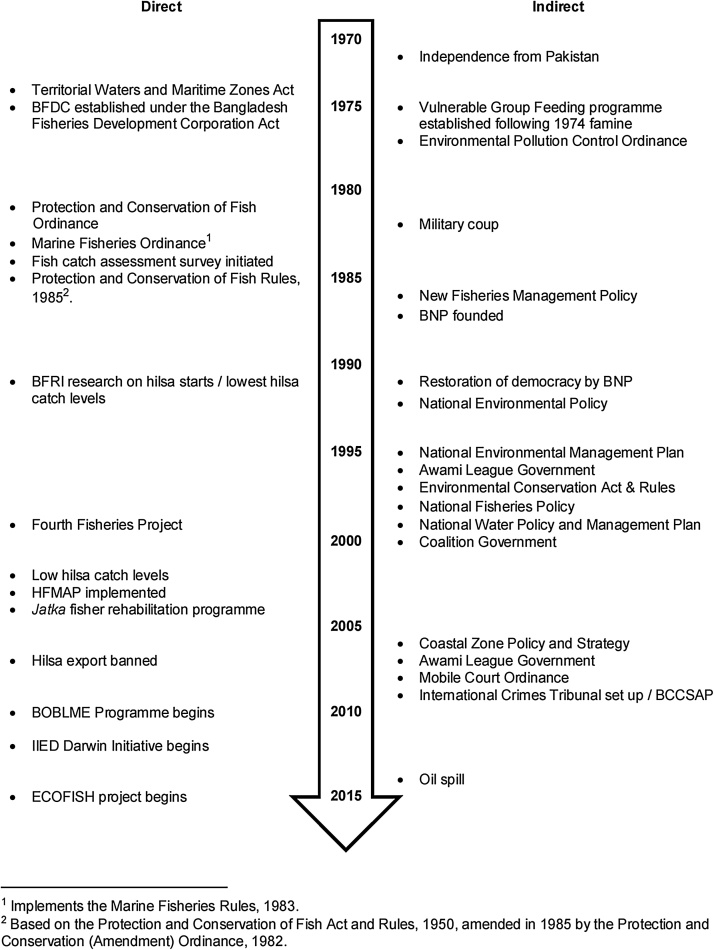
Timeline of key institutional, social, economic, and environmental events in the recent history of Bangladesh classified by potential direct and indirect relevance for hilsa. BFDC = Bangladesh Fisheries Development Corporation; BFRI = Bangladesh Fisheries Research Institute; BNP = Bangladesh Nationalist Party; HFMAP = Hilsa Fishery Management Action Plan; BOBLME = Bay of Bengal Large Marine Ecosystem; IIED = International Institute for Environment and Development; BCCSAP = Bangladesh Climate Change Strategy and Action Plan. Cyclones, famines and other natural disasters are not presented here because of their high frequency. Extra details can be found in mmc2 (in Supplementary material).

First, we took a historical perspective and compiled the key institutional, social, economic and environmental events in the recent history of Bangladesh. We selected these events according to their potential direct or indirect relevance to the hilsa fishery, which we established through literature review and key informant interviews (Bladon, 2016). The resultant figure (Fig. 1) provides a timeline that can be used for reference throughout the results section. Bull et al. (2015) set the context of their frame of reference with 100 years of history, since this is approximately how long Uzbekistan had existed as a defined international entity. We therefore restricted our analysis of Bangladesh to the years since its independence in 1971 – approximately 50 years of history.

### 2.2 Frame of reference

To develop the frame of reference, we explicitly considered trends in potential physical, social, economic, and institutional drivers of ecological change in the hilsa fishery, before exploring trends in hilsa abundance and distribution – scanning the available literature and analysing secondary data compiled from published literature, online sources, and collaborators at the Bangladesh Department of Fisheries (DoF). This allowed us to establish a current baseline for the hilsa fishery, structured by each of the four categories in our framework and presented in the context of historical change over the last 50 years.

Based on expectations formed through this analysis, we developed a conceptual map of the potential interactions between these drivers, within and between categories. We outlined the key expected interactions between hilsa and drivers of change (i.e. those most relevant to the sustainability of the hilsa fishery) and created two potential counterfactuals, consisting of projections based on these trends and interactions. Together, the baseline and counterfactuals form the frame of reference. Various potential counterfactuals could be projected from the trends presented in this study. We chose two feasible extremes to illustrate the range of potential outcomes, representing the ‘most desirable’ and ‘least desirable’ scenarios from the perspective of the sustainability of the fishery. Although this entailed making an implicit value judgement about what is and is not desirable for the hilsa fishery, it also allowed us to make our assumptions explicit. We interrogated the differences between the two potential counterfactuals to identify outstanding areas of uncertainty and their associated management implications. Finally, we highlighted information needs to improve the robustness of the frame of reference as a tool for action and intervention evaluation.

#### 2.2.1 Selection of potential drivers of change

Based on our understanding of hilsa biology, and that of similar species, we identified factors that are known to or would be expected to a) directly affect hilsa populations, and b) indirectly affect hilsa populations. This selection process relied on extensive literature review and interviews with key stakeholders (Bladon, 2016). For each potential driver, we reviewed the literature and assessed secondary datasets for trends, conducting statistical analysis where possible. Here we provide a brief justification for selection of drivers in each category.

##### 2.2.1.1 Physical drivers

The life history of hilsa is known to be influenced by environmental conditions including water salinity, turbidity, flow, temperature, pH, dissolved O^2^, and phytoplankton availability (Ahsan et al., 2014; Rahman et al., 2012a). We therefore identified key physical drivers that have been linked to general fish abundance and distribution, to changes in those environmental conditions, or directly to the abundance and distribution of hilsa. These drivers were: climate change, water diversion activities, forest cover, and pollution (see mmc1 in Supplementary material).

##### 2.2.1.2 Social drivers

We identified human population and poverty to be drivers of change in the hilsa fishery (see mmc1 in Supplementary material). Human population size influences the extent of fishing pressure in Bangladesh, because capture fisheries are essential for livelihood support and food security (Belton et al., 2014; FAO, 2014). Human population size also influences the extent of development and infrastructure, including agriculture and aquaculture, which place pressure on hilsa habitat (BOBLME, 2011). In hilsa fishing areas, low income is associated with fishing juvenile hilsa (known in Bangladesh as *jatka*) and with strong fishing dependence, which is characterised by illegal fishing and lack of other livelihood activities (Bladon et al., 2018).

##### 2.2.1.3 Economic drivers

We identified the primary economic activities in Bangladesh, and those which are fast-growing or being explored, and assessed trends in these activities. All of these activities are relevant to the hilsa fishery through the impacts of livelihood availability and wealth on fishing pressure (Bladon et al., 2018). Some of them also have direct or indirect environmental impacts on hilsa populations: agriculture and aquaculture can affect hilsa habitat through eutrophication (BOBLME, 2011): factories can contaminate rivers (Karn and Harada, 2001); and mechanised vessels, shipbuilding, ship breaking, and oil and gas extractive industries can pollute coastal waters (Chowdhury et al., 2017; Hossain et al., 2016). Because of the direct impact of exploitation on hilsa abundance, we focused largely on bioeconomic trends in the fishing industry, looking for trends in commercial catch per unit effort (CPUE) and economic value in the hilsa fishery.

##### 2.2.1.4 Institutional drivers

The effectiveness of fisheries management is a crucial determinant of sustainable fisheries. Management effectiveness depends on a range of factors, including: a robust scientific basis for management recommendations, transparency in turning recommendations into policy, and capacity to enforce and ensure compliance with regulations. We reviewed the current national institutional and legislative context of fisheries management, exploring how it has changed in recent history, with a focus on hilsa fishery management. We also evaluated the current institutional framework, with respect to scientific rigor, capacity, and transparency.

#### 2.2.2 Hilsa trends

Although categorised as a species of least concern (Freyhof, 2014), hilsa – like many other shads – are poorly understood, particularly in their marine phase (Bladon, 2016). We explored trends in abundance and distribution of hilsa by compiling all available information on the key rates that partially determine this abundance and distribution (movement, reproduction, growth, and mortality). We also explored trends in stock status; due to data limitations, no reliable stock assessments have been conducted in Bangladesh waters, but we reviewed the available information. We included official national catch statistics (and CPUE, where available), which can be useful indicators of abundance when other data are sparse (Pauly, 2013).

## 3 Results

### 3.1 Brief recent history

The People’s Republic of Bangladesh gained independence from Pakistan at the end of the Liberation War in 1971 and has since experienced rapid political, socioeconomic, and environmental change (Fig. 1). Following independence, Bangladesh suffered from famine, natural disaster, and military rule, before the restoration of parliamentary democracy in 1991. Since then, power has passed from the Bangladesh Nationalist Party to the Awami League. Due to widespread corruption, institutional politicisation, and misallocation of resources, governance is poor (BTI, 2012). This has allowed developmental NGOs – through their partnership with western aid agencies – to dominate the rural economy and act as a powerful ‘shadow state’ (Karim, 2008). Political tensions remain and, following the creation of the International Crimes Tribunal in 2009, tensions and violence led to the disruption of transport and movement of goods (BTI, 2012). Nevertheless, Bangladesh overperforms on social development indicators, such as health, sanitation, and education, when compared to countries with a similar level of per capita income, and has consequently become a model for other developing countries (Asadullah et al., 2014).

Numerous environmental acts and supporting policies have been introduced during the process of rapid industrialisation and urbanisation triggered by independence – many of which are directly or indirectly relevant to the hilsa fishery (see Section 3.5.1 for fisheries policies). For example, the National Environmental Conservation Rules, introduced following the 1995 Act, emphasised the mitigation of industrial water pollution; and the Bangladesh Climate Change Strategy and Action Plan, developed in 2009, has largely succeeded in mainstreaming climate change into national and sectoral development planning (Huq, 2010). However, in general, legislation has not kept pace with environmental change and it is often poorly implemented (BOBLME, 2011; Clemett, 2004).

### 3.2 Trends in physical drivers

#### 3.2.1 Climate change

As a low-lying deltaic country, Bangladesh is extremely vulnerable to the impacts of climate change (Huq, 2001). It suffers from intense tropical cyclones and storm surges and although there has been an emphasis on climate change adaptation research in recent years, there have been few regional climate change studies focusing on Bangladesh and the country lacks capacity for implementation (Agrawala et al., 2003; Reid and Huq, 2014). Linear models showed a significant upward trend in temperature, and a significant downward trend in rainfall during the period 1983–2014 (see mmc2 in Supplementary material).

#### 3.2.2 Water diversion activities

The construction of dams and barrages for irrigation and flood control within and outside Bangladesh, together with estuary drainage and land reclamation projects, have led to hydrological and morphological changes in rivers, and probably increased salinity downstream (see mmc2 in Supplementary material).

#### 3.2.3 Forest cover

Recent net annual change in forest cover appears to be stable or even positive in Bangladesh, but mangrove loss has been caused by timber collection and by increased salinity resulting from unregulated encroachment of shrimp farming, storm surges, and reduced freshwater flow (see mmc2 in Supplementary material).

#### 3.2.4 Pollution

River pollution from industrial effluents and untreated municipal waste and sewage, eutrophication, and petrochemical pollution in coastal waters are widespread and growing problems in Bangladesh (see mmc2 in Supplementary material).

### 3.3 Trends in social drivers

The population of Bangladesh has been growing since before independence, particularly in urban areas (UN, 2015, 2018). Extreme poverty is prevalent in the coastal areas where hilsa fisheries are concentrated, and fishers are often described as the poorest, most vulnerable social group in the country. However, there has been a steady decline in poverty over the last decade or so (see mmc2 in Supplementary material).

### 3.4 Trends in economic drivers

The economy of Bangladesh is rapidly developing, largely through industrialisation and exports. It had an average national GDP growth rate of six per cent over the decade to 2012 (BBS, 2012) and is classified as one of the ‘Next Eleven’ – a group of countries recognised for their potentially large, fast-growing markets (Goldman Sachs, 2007). Industries of significance include clothing, shipbuilding and ship breaking, and agriculture – which has modernised and mechanised in recent years (mmc2 in Supplementary material).

#### 3.4.1 Fishery sector

**Fig. 2:**
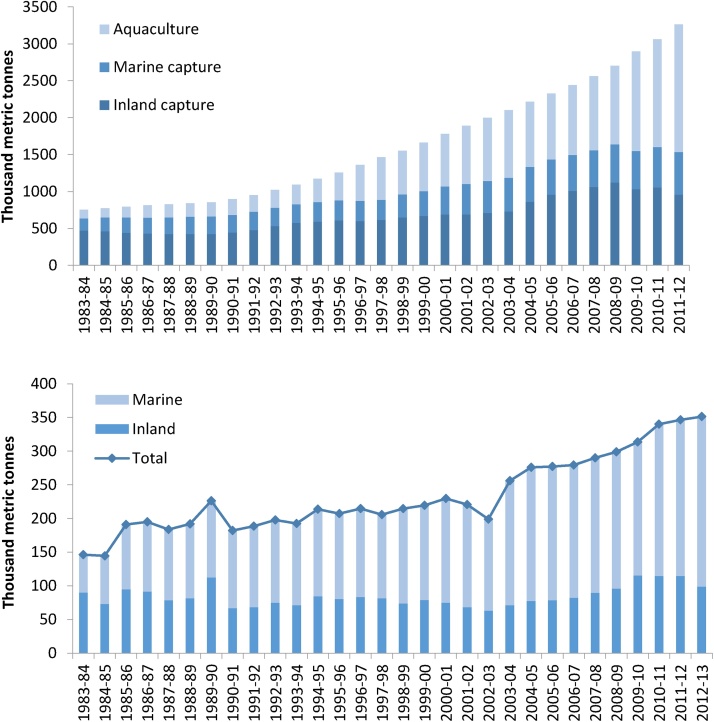
**(a)** Total reported marine and inland fishery and aquaculture production in Bangladesh from 1983-84 to 2011–2012; and **(b)** Total reported annual hilsa landings, inland hilsa landings and marine hilsa landings in Bangladesh from 1982–1983 to 2012–2013 (DoF, 2014).

Bangladesh is heavily reliant on its inland and marine fisheries for trade, as well as subsistence. They contribute over four per cent to the national GDP and are second only to the garment industry in foreign exchange earnings, which are derived largely from shrimp and prawn exports (FRSS, 2013). There has been a general upward trend in total fish production since 1983 (Fig. 2), and according to production statistics (DoF, 2014), the total fishery sector growth rate more than doubled between 2005 and 2012. A steady increase in aquaculture production contrasts with a decline in capture fishery production since 2008 (Fig. 2). By 2012, aquaculture had become the dominant source of fish production (contributing over 50%), while small-scale inland capture fisheries contributed about 30% and marine capture fisheries 18%. An estimated 88% of marine catches were attributed to artisanal fisheries, and the remainder to the industrial (trawl) sector (FRSS, 2013). The reliability of these statistics is, however, questionable; a reconstruction of marine fisheries catches for Bangladesh from 1950 to 2010 found reconstructed catches to be 157% higher than those reported by Bangladesh to the FAO, largely due to unreported subsistence catches (Ullah et al., 2014).

#### 3.4.2 Hilsa fishery characteristics

Hilsa are distributed throughout coastal regions of the Indian Ocean, from Kuwait eastwards towards Myanmar (Whitehead, 1985), but stocks in the Persian Gulf are genetically distinct from those in the Bay of Bengal (Milton and Chenery, 2001; Salini et al., 2004). Bangladesh lands 50–60% of reported global hilsa catches (BOBLME, 2010). The hilsa fishery is a commercial one comprised of inland (artisanal) and marine (artisanal and industrial) sectors; hilsa are rarely caught purely for subsistence because they are high value fish (Ullah et al., 2014). Fishing occurs throughout the year, peaking from mid-August to October, when the majority of hilsa are reportedly caught (60–70%), and to a lesser extent in January and February, inland (Rahman et al., 2012b). Fishing activity is reduced in most areas from November to July, particularly during the monsoon months from June to mid-August.

Most hilsa catch is marketed and consumed domestically as fresh fish (Alam, 2010), but post-harvest management of fish in Bangladesh is generally poor. Only Bangladesh Fisheries Development Corporation (BFDC) landings centres at Cox’s Bazar and Chittagong (established specifically for the marketing of BFDC trawler catch) have adequate facilities (Ahmed, 2007; Islam, 2003), but there are about 6500 private fish markets in the country, more than half of which are small village markets (Islam et al., 2016). Post-harvest loss rises during peak fishing season, when supply exceeds the availability of block ice for preservation (see mmc2 in Supplementary material). Until the late 2000s less than two per cent of total catch was legally exported to India and countries in the Middle East, Far East, Europe, USA and Australia where there are Bangladeshi diaspora, bringing in some foreign exchange earnings (Table B.2 in Supplementary material mmc2; Alam, 2010; Mome and Arnason, 2007). However, the DoF has since implemented an export restriction (2007) and ban (2012 onwards) – a reported attempt to reduce the domestic price of hilsa and increase availability of the fish in national markets, with probable political motivations (Padiyar et al., 2012; M. Mome, Department of Fisheries, personal communication, 29th May 2014). In fact, a great deal is still being illegally exported to Kolkata (C. Meisner, WorldFish, personal communication, 26th May 2014).

#### 3.4.3 Hilsa catch and effort monitoring

In 1983, the DoF initiated an annual catch assessment survey system for inland and marine fisheries in Bangladesh, with an emphasis on hilsa. There are, however, problems with sample sizes and sampling procedure: The assessment focuses on sample villages (many of which no longer exist) in major rivers and some marine areas, and it cannot therefore provide an accurate picture of catches. A reconstruction of marine catches from 1950 to 2010 bears out this lack of reliability, indicating that hilsa makes up 18% of total marine catches and 41% of artisanal marine catches, and that reported landings have been underestimated by 19% (Ullah et al., 2014).

The contribution of hilsa to total reported capture fishery production, estimated from the catch survey, increased from 18% in 1983 to 30% in 2012 (Table B.1 in Supplementary material mmc2). Historically, fishing was concentrated upstream in major rivers, but the proportion of hilsa landings coming from the inland fishery has apparently declined, and over the last two decades the marine sector has become the dominant reported source of hilsa (Fig. 2). This shift has been attributed both to *jatka* overfishing inland and to the mechanisation and expansion of the marine sector (Amin et al., 2002; BOBLME, 2010; Haldar, 2004).

The lack of reliable estimates of annual fishing effort makes actual CPUE difficult to calculate, particularly for the inland fishery. Numbers of mechanised and non-mechanised boats in the artisanal marine sector have been estimated by the DoF and appear to have increased since 1983, which indicates that numbers of fishers (although not specifically hilsa fishers) are also increasing (Haldar, 2004; Table B.3 in Supplementary material mmc2). Analysis of official marine landings and effort data shows a decline in CPUE between 1984 and 2006, although the trend was less clear for the non-mechanised sector (Mome and Arnason, 2007; Sharma, 2012). Sharma (2012) found catchability (the relationship between CPUE and standing biomass) of hilsa in the non-mechanised marine sector to be almost twice that of the mechanised marine sector – probably because the mechanised sector is targeting other species as well (rather than just targeting vulnerable concentrations of migrating hilsa) and it has a shorter history of fishing.

Catch values are also difficult to estimate because of the complexity of the market chain and the relationships between fishers and intermediaries (Porras et al., 2017; mmc2 in Supplementary material). Estimated annual landings revenues range from USD 380 million (or one per cent of GDP) in 2006 to USD 640–850 million in 2009 (Mome and Arnason, 2007; Sharma, 2012). By multiplying an average value of 430 BDT per kg (based on a one-year survey of four fish markets; Fernandes et al., 2015) by the latest reported annual hilsa catch volume, we arrived at a much higher estimate of two billion USD. Through a very simple bio-economic assessment of the marine artisanal fishery (restricted by data limitations), Mome and Arnason (2007) found annual net profit to be only seven per cent of total revenues, due to high costs of fishing. Concluding that stocks were overexploited, they made recommendations for a 60% reduction in effort, through restrictions on numbers of mechanised vessels in the artisanal marine sector. In turn this was expected to more than double hilsa stocks and increase individual vessel catch rates, which they calculated could raise annual net profits by 10–15%. Yet, the industrial sector, in contrast to the artisanal sector, has been described as under-developed and under-utilised, and Bangladesh is now aiming for a ‘blue economy’ under which the trawler industry could expand (C. Meisner, WorldFish, personal communication, 26th May 2014).

### 3.5 Trends in institutional drivers

Fisheries management in Bangladesh has been criticised for corruption at various levels, and a lack of resources and general institutional weakness has contributed to low levels of compliance (Ali et al., 2010; Bladon, 2016; Islam, 2003; Jentoft and Onyango, 2010). Bangladesh has been ranked 47^th^ out of the 53 most active fishing countries in the world for compliance with the UN Code of Conduct for Responsible Fisheries (Pitcher et al., 2009), and the effectiveness of its marine fisheries management regime has been scored among the lowest in the world (Mora et al., 2009). Fig. 1 provides a timeline of fisheries policy and research in Bangladesh, the details of which are provided in mmc2 (in Supplementary material), together with a summary of administrative hierarchy.

#### 3.5.1 Hilsa management

Government budget allocations to the DoF for hilsa management increased from about USD 4.11 million in 1998–1999 to USD 23.11 million in 2014–2015 (Majumder et al., 2015a). The DoF responded to concerns about declining hilsa catches in 1991 by initiating research on the fishery, which led to international collaborations with the Australian Centre for International Agricultural Research (ACIAR) and the World Bank-DFID Fourth Fisheries Project (FFP). Policy directives and recommendations from these projects have largely been implemented through the Hilsa Fisheries Management Action Plan (HFMAP). More recently, research has been conducted on hilsa through a Darwin Initiative project led by the International Institute for Environment and Development (IIED) and the WorldFish Enhanced Coastal Fisheries (ECOFISH) project, which is supporting the DoF to develop an updated HFMAP. Based on the assumption that recruitment is compromised, and that this is a result of overfishing *jatka* and spawning hilsa, the HFMAP aims to sustain and increase hilsa production, prevent loss of hilsa habitat and build the capacity of implementing organisations (DoF, 2002). Several key recommendations have been taken up and implemented as acts, ordinances, rules and fisheries management initiatives (Fig. 1), many of which are intended to support the artisanal hilsa fishery directly, largely through the protection of *jatka* (formally defined as hilsa less than 25 cm in length). These are summarised below (see mmc2 in Supplementary material for more detail). A comprehensive assessment of the impact of management is beyond the scope of this paper, but weaknesses have been identified in the institutional arrangements behind these policies, which lead to poor enforcement of rules and limit their effectiveness (Bladon, 2016; Bladon et al., 2016).

##### 3.5.1.1 Protection of spawning hilsa

**Fig. 3:**
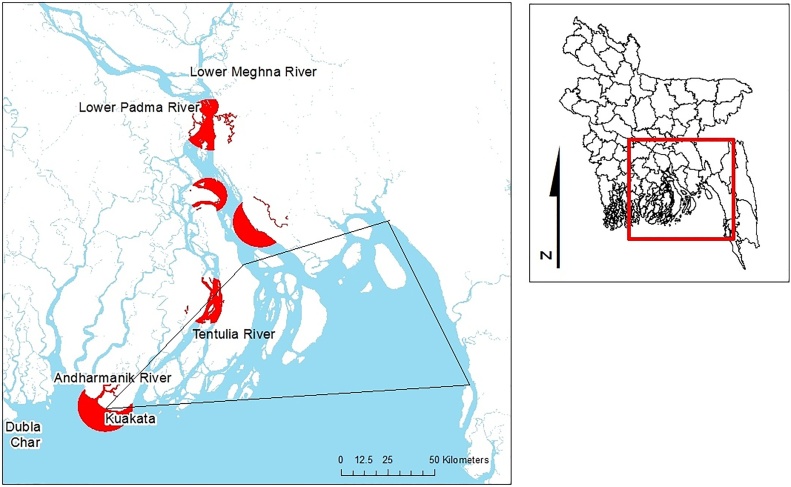
Map showing sanctuary areas (red) and rivers flowing into the Bay of Bengal (blue). From north to south the sanctuaries are: 100 km of Meghna River from Chandpur to Laxmipur, 20 km of Padma River in Shariatpur, 90 km of Shahbajpur channel (Meghna tributary), 100 km of the Tentulia River from Bhola to Patuakhali districts, and 40 km of the Andharmanik River in Patuakhali. All fishing is banned in the sanctuaries from March to April, apart from the Andharmanik River, where fishing is banned from November to January. Black polygon demarcates important spawning area where enforcement is targeted during peak spawning season (For interpretation of the references to colour in this figure legend, the reader is referred to the web version of this article).

Spawning hilsa are protected with a ban on hilsa fishing throughout the country for 15 days of the perceived peak breeding season, with the aim of minimising disturbance to spawning and recruitment (Bladon, 2016). Monitoring for compliance with this ban is targeted within a 7000 km^2^ area that is thought to cover important spawning grounds (Fig. 3).

##### 3.5.1.2 Implementation of protection and Conservation of Fish Act and Rules, 1950 (MoFL, 1985)

This includes a nationwide ban on all activities related to *jatka* between 1st November and 31st July, and a ban on use and production of monofilament gillnets. Enforcement activities are targeted to the 152 sub-districts where *jatka* are distributed, and particularly those areas that are thought to be important nursery grounds.

##### 3.5.1.3 Hilsa sanctuaries

Under the amended Fish Act and Rules (MoFL, 1985), four major nursery areas in the Meghna and Padma rivers were designated hilsa sanctuaries in 2005, with another designated in the inshore marine area in 2011 (Fig. 3). All fishing is banned in these areas during their perceived peak period of *jatka* presence: March to April in all but the southernmost sanctuary (a 40 km stretch of the Andharmanik River where fishing is banned from November to January).

##### 3.5.1.4 Jatka fisher rehabilitation

In 2004 the DoF introduced the *jatka* fisher rehabilitation programme, which aims to improve the socioeconomic condition of affected fishers living inside and around sanctuary areas and thereby to incentivise compliance with the fishing bans (Islam et al., 2016). This is largely based on the distribution of compensation in the form of rice during ban periods (centered on the sanctuary fishing bans), with some alternative livelihood support and awareness raising activities.

##### 3.5.1.5 Marine fishing ban

In 2015 an amendment was made to the Marine Fisheries Ordinance and Rules (MoFL, 1983), which bans all fishing by all vessels in the marine fisheries between May 20th and July 23rd each year, for conservation purposes. No robust data have been published to justify this ban, but the DoF has based it on concern for hilsa populations (Bladon, 2016).

##### 3.5.1.6 Regional hilsa management

Although Bangladesh shares its hilsa stocks with other countries in the Bay of Bengal, there is no formal agreement in place for regional management of hilsa (Rahman et al., 2012b). However, the Bay of Bengal Large Marine Ecosystem project (BOBLME) is supporting countries in the implementation of an ecosystem-based approach to the management of shared Bay of Bengal fish stocks, under which a Hilsa Fisheries Assessment Working Group has been established to provide technical information to the Bay of Bengal countries (BOBLME, 2014). The IUCN has also proposed a set of policy options for transboundary management of hilsa for India and Bangladesh (Ahsan et al., 2014), and there has been a recent push to strengthen regional cooperation generally (BBS, 2012).

##### 3.5.1.7 Hilsa culture

As an alternative approach to increasing wild hilsa production, the MoFL has been supporting the exploration of pond culture (not found to be economically viable) and cage culture, which has had limited success (Puvanendran et al., 2013; Sahoo et al., 2016). Captive breeding techniques are also being explored to supplement the natural population levels through ‘hatch and release’ (BOBLME, 2014).

### 3.6 Trends in hilsa

Details of the key rates that partially determine hilsa abundance and distribution (movement, reproduction, growth, and mortality), as well as their trends, can be found in mmc3 (in Supplementary material). Here we summarise trends in those rates, as well as overall trends in hilsa abundance and distribution, including stock status. Hilsa range from the Bay of Bengal to the rivers of Bangladesh, India and Myanmar, and this range may have been reduced (Blaber et al., 2003; Hasan et al., 2016; Miah, 2015). Understanding of migratory routes and the habitat types suitable for each life history stage is incomplete (Ahsan et al., 2014; Bhaumik, 2015), and marine and inland populations have received limited individual research attention; whereas commercial CPUE data are available for marine populations but not inland populations, marine populations have received much less experimental research attention than inland populations (Rahman et al., 2012b; Sharma, 2012). From the data available, there appears to have been an increase in natural mortality, fishing mortality and exploitation rates, and a decline in size, maximum yield-per-recruit, and marine CPUE (Rahman and Cowx, 2008; Sharma, 2012). However, no data have been published beyond 2009, and much of this information is conflicting or limited in reliability, so trends in abundance are still unclear. While some stock assessments have concluded that hilsa are overfished, exploitation rates are highly uncertain and other risk assessments suggest that populations – although vulnerable to overfishing – may not yet be in decline (Amin et al., 2004; Mome and Arnason, 2007; BOBLME, 2010; Sharma, 2012; Bala et al., 2014).

### 3.7 The frame of reference

#### 3.7.1 Baseline

The baseline for this study indicates a complex, understudied, and vulnerable fishery in a rapidly developing country which suffers from technical and institutional gaps and numerous environmental threats (Table 1; mmc2 in Supplementary material).

**Table 1:** Trends and current baseline conditions for hilsa in the context of drivers of change, alongside historical change over the last 50 years (bioeconomic drivers refer specifically to hilsa fishing and are discussed within Section 3.4).

Driver	Trends over recent history	Current baseline
Physical	• Warming temperatures, reduction in annual precipitation, probable intensification of monsoon precipitations, sea-level rise • Increased water diversion activities reduce inputs of freshwater and silt • Water pollution has increased in inland and coastal areas • Forest cover declined dramatically but recently stabilised	• Changes in quality and availability of hilsa habitat and decline in river flows have likely led to a decline in range and abundance of hilsa (particularly inland), and disruption of migratory routes • But reported impacts of the trends presented here on hilsa are largely conjecture, and potentially difficult to tease apart

Social	• Human population has grown • Average poverty levels have declined, though more slowly in fishing-dependent regions • Hilsa have long had a strong cultural importance, but *jatka* were historically the more affordable option • Traditionally, coastal fishers were ‘low caste’ Hindus, but increasing numbers of poor and landless Muslims are fishing and wealthy Muslims are investing	• Widespread illegal fishing activities linked to poverty and cycles of debt

Economic	• Rapid industrialisation and urbanisation	• Uncertain impacts of economic trends, but probably affecting hilsa populations through impacts on physical habitat

Bioeconomic	• Contribution of hilsa to total fishery production has increased, largely through an increase in marine production • Marine CPUE has declined • Foreign exchange earnings from hilsa reduced to zero due to export restrictions and ban • *Jatka* are caught mainly inland, increasingly with monofilament nets • Inland landings have remained fairly constant, but inland CPUE is undocumented	• Hilsa fishery has a low annual net profit • Production is dominated by marine sector • Inland sector may be overfished, but evidence is largely anecdotal

Institutional	• Introduction of various management measures by the state to increase hilsa production and protect habitat, with a focus on jatka conservation.	• Poor monitoring and enforcement of fishing regulations • Biological justification for management limited by lack of reliable stock assessment • No international regional management

#### 3.7.2 Interactions

**Fig. 4:**
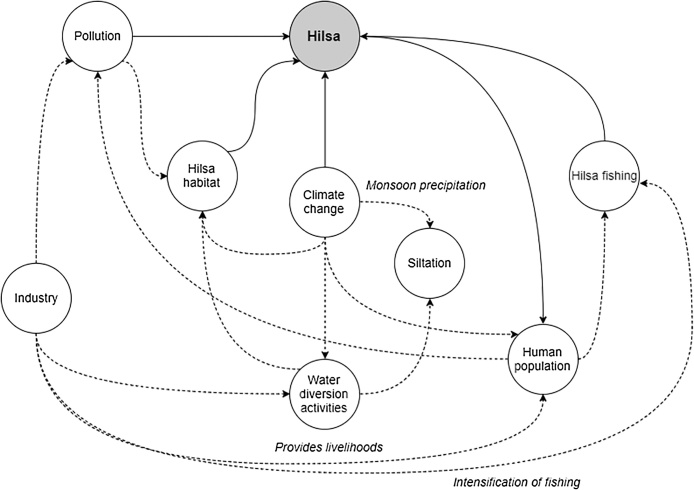
Conceptual map of potential factors key to hilsa conservation interventions in Bangladesh.

Current understanding of hilsa ecology indicates that any fluctuation in hilsa populations could result from a combination of institutional, economic, social and physical factors, which may compound each other and interact in myriad ways (Fig. 4). Aside from fishing, key potential drivers of change in hilsa are the physical factors which interact to determine juvenile and adult habitat quality and availability, both of which appear to be declining (BOBLME, 2010). For example, climate change, water diversion activities, and related siltation may interact to block migratory routes, and pollution may interact with deforestation, climate, and water diversion activities to reduce primary production and water quality. Most of these factors have multiple routes of potential impact. For example, in addition to direct mortality or reduction in habitat quality, pollution could also undermine current management interventions; if sanctuary areas are polluted, then the efficacy of temporal closures will be severely reduced. Another example is the reduced freshwater discharge and increased siltation resulting from water diversion activities; at the same time as reducing spawning grounds and reducing the suitability of habitat in terms of depth, salinity and turbidity, it may be concentrating migrations, making hilsa more vulnerable to being caught (Miao et al., 2010; Rahman et al., 2012a). While these diversion activities may reduce downstream siltation in some areas, sediment loading is closely coupled with monsoon inundation, so it is possible that intensification of this monsoon could be contributing to increased sediment loads elsewhere, reducing habitat suitability for hilsa (Goodbred and Kuehl, 2000; SRDI, 1998). On the other hand, dredging activities could be reopening some river channels for migration.

There are several positive and negative feedback loops at work involving socioeconomic change. In addition to direct impacts of climate change on hilsa feeding, spawning, and migratory behaviours, changes in chemical and physical parameters of habitat could negatively impact hilsa populations via an increase in human vulnerability and poverty and therefore dependence on *jatka* in coastal areas (Adger et al., 2003; Dasgupta, 2014), or through increased demand for water diversion activities. However, positive impacts may also arise through urbanisation and human migration away from coastal areas, which could reduce artisanal fishing pressure (BBS, 2009; GED, 2012). Although industrialisation has led to a reduction in poverty, this does not seem to have led to a reduction in numbers of artisanal hilsa fishers. In fact, increased mechanisation of vessels may have increased pressure on the fishery, and with urbanisation and industrialisation comes pollution: a rise in the numbers of vessels and oil spills in the Bay of Bengal is causing petrochemical pollution, and as the recent oil spill on the south west coast of Bangladesh demonstrated, the country has little contingency for such disasters (BOBLME, 2011; Islam et al., 2017).

The complex interactions between hilsa fishing, institutional context, and ecological trends are beyond the scope of this paper. If the rationale underpinning current hilsa management and its implementation is sound, it should in theory protect against the overfishing of *jatka* and adults and thereby influence trends in hilsa abundance and distribution. However, the scientific basis for current management is often weak and there is evidence to suggest that implementation suffers from enforcement and compliance issues (Bladon, 2016; Bladon et al., 2016).

#### 3.7.3 Counterfactuals

Through analysis of the trends in key drivers of the sustainability of the hilsa fishery, and the potential interactions between these drivers, we now identify two potential counterfactuals (i.e. projected trends under different scenarios; Table 2). Below we describe these projections, focussing on three areas of change and uncertainty which our analyses suggest are of particular importance to hilsa management and conservation going forward.

**Table 2:** Projected feasible outer-bound counterfactuals for hilsa in the context of trends in drivers of change.

Driver	Undesirable counterfactual	Desirable counterfactual
Institutional	• No effective change in institutional arrangements	• Protection of hilsa increases through improved monitoring and enforcement
		• Development of regional hilsa management plan
		• Fishery closures adapt to keep pace with environmental change

Social	• Poverty is slow to decline in coastal areas and illegal fishing continues, with no reduction in dependence on fishing	• *Jatka* fisher rehabilitation programme and job creation help to reduce illegal fishing and dependence on fishing

(Bio)economic	• Expansion of the artisanal fishery causes a decline in production and stock collapse within one decade	• Stable or reduced effort in the artisanal fishery slows a decline or stabilises production
	• Expansion of industrial fishery limited by lack of enforcement capacity	• Expansion of industrial hilsa fishery sustainable due to reduced artisanal fishing
	• No advances in hilsa cage culture or captive breeding	• Development of cage culture and captive breeding techniques reduces pressure on wild populations
	• Expansion of polluting industries	• Pollution prevention programmes mitigate some negative impacts of industry on hilsa in the long term
	• Climate change and political unrest limits economic development	• Economic development leads to job creation and urban migration
	• Existing power structures remain and continue to limit profitability to fishers	• Improved access to financial products increases profitability to fishers

Physical	• Water diversion activities, climate change, siltation and pollution disrupt migratory routes and reduce habitat quality	• Improved implementation of fisheries and environmental policies mitigates some disruption of migratory routes in the short term, but the long-term impacts of climate change on habitat quality are unavoidable
	• Climate change may affect feeding, spawning and migratory behaviour via physical and chemical parameters	• No significant shift in behaviour is caused by climate change

##### 3.7.3.1 Climate change

Bangladesh is likely to continue to experience climate change, with an overall reduction in precipitation and an increase in monsoon precipitation (Adger et al., 2003; Agrawala et al., 2003; Rahman et al., 2012c). Assessments of the potential impact of climate change on cyclone frequency and intensity in Bangladesh are tentative (Karim and Mimura, 2008), but the IPCC has estimated a 5–10% increase in peak intensity and a 20–30% increase in associated precipitation rates (IPCC, 2013), the effects of which would be compounded by projected global sea-level rise (Huq, 2001; Karim and Mimura, 2008). Model projections show a steady rise in sea surface temperatures in Bangladesh over the next century, with a potential increase in net primary production (Fernandes et al., 2015).

Climate change is expected to cause a decline in fish production potential in South Asian countries that are highly dependent on fisheries, and specifically in Bangladesh (Barange et al., 2014; Habib et al., 2014). Modelling indicates that these changes will cause a decline in marine hilsa production potential in Bangladesh over the course of the century (Fernandes et al., 2015), although, within Bangladesh there may be some habitats that become more suitable for hilsa and others that become less suitable. Changes in water temperature, turbidity, nutrient levels and salinity are likely to impact migratory behaviour and therefore restrict hilsa distribution – especially when considered in combination with the possible effects of water diversion activities, associated siltation, land reclamation, and pollution. Without habitat restoration, and depending on future rates of deforestation, submerged islands in downstream areas are likely to continue blocking migratory paths (DoF, 2002) and spawning behaviour might also be affected by warming and changes in freshwater flow. Although marine and inland populations are inextricably linked, inland populations are assumed to be more vulnerable to the impacts of physical drivers and this is where depletion will likely occur first.

##### 3.7.3.2 Human population growth

The human population of Bangladesh is projected to continue growing until around 2060 and the current majority rural population is expected to shift to majority urban (UN, 2015, 2018). The Government’s target for an annual economic growth rate of 10%, based on accelerated growth in exports and remittances, would take Bangladesh across the middle-income country threshold by 2021 and lead to a national decline in poverty (GED, 2012; UN, 2015). Yet, it should be noted that continued political unrest and projected climate change (through increased frequency and intensity of natural disasters) could hinder economic growth (EIU, 2015). Without the introduction and enforcement of more effective pollution prevention practices in Bangladesh, water pollution is expected to worsen with continued industrialisation and population growth (BBS, 2012; Hoque and Clarke, 2013). Rivers will be subject to increased waste generation, effluents from the garment industry – which is projected to continue growing (CIA, 2014) – and eutrophication caused by the intensification of agriculture and aquaculture (BOBLME, 2011). Petrochemical pollution is expected to negatively impact hilsa populations in the Bay of Bengal through increased numbers and continued mechanisation of vessels, growth of the shipbuilding and shipbreaking industries, and potential expansion of the oil and gas sector.

Together with economic drivers of urbanisation, projected changes in climate are expected to trigger continued migration of human populations away from coastal areas towards cities (BBS, 2009). But climate change also increases poverty and vulnerability (Adger et al., 2003; Dasgupta, 2014) and, since rural coastal communities in the west of Bangladesh are expected to be the slowest to rise above poverty, dependence on fishing may remain high and *jatka* fishing may continue or even increase. These impacts will depend partly on local adaptation responses (Hobday et al., 2016).

##### 3.7.3.3 Fisheries management

In the absence of any institutional improvements, modelling suggests that increased levels of fishing effort on adult or *jatka* populations could lead to a collapse of hilsa stocks in Bangladesh within one to two decades (Bala et al., 2014; Fernandes et al., 2015; Mome and Arnason, 2007; Sharma, 2012). Given the Government’s aim to increase hilsa production, and given industry trends, the marine fishery – both artisanal and industrial sectors – looks set to continue expanding (DoF, 2002). This expansion would require improvements in post-harvest technology and infrastructure, which could lead to overexploitation in the Bay of Bengal (Habib et al., 2014). Even with these improvements, it is unlikely that the export ban will be lifted, and so expansion of the export market is unlikely.

Total aquaculture production will almost certainly continue its upward trend, although the Government is mindful of maintaining its capture fisheries (Fernandes et al., 2015). Currently hilsa is not suitable for aquaculture. Even if substantial progress is made with economically viable hilsa cage culture, it would be unlikely to take much pressure off the wild capture hilsa fishery, given the social and cultural importance of and demand for hilsa. Recommendations have been made for effort restrictions on the artisanal fishery, but given the weak enforcement capacity these are probably much less realistic than the current spatial and temporal fishing bans (Mome and Arnason, 2007). If a major threat to *jatka* and spawning adults is indeed overfishing, then improved monitoring and enforcement of current management rules could prevent collapse and potentially lead to increased hilsa abundance (Bladon, 2016). Sharma (2012) pointed out that since intrinsic growth rates are high, hilsa should respond quickly to conservation interventions. Projections indicate that with more sustainable management, some climate change impacts on marine hilsa production could be mitigated, although it would not halt a long-term decline (Fernandes et al., 2015). However, in light of the projected development of the marine fishery, the efficacy of these rules, which are enforced largely inland, is uncertain. The proposed development of a regional hilsa fishery management programme should reduce the impacts of physical drivers or fishing pressure from adjacent countries, but it is ‘only an idea’ (M. Khan, Bangladesh Fisheries Research Institute, personal communication, 19th May 2014).

At the time of writing, a Conservation Trust Fund (CTF) was going through the ratification process, with the aim of generating financial resources for hilsa conservation (Majumder et al., 2015b). Although the proposed CTF does not conform to best practice standards in terms of governance structure, it could be used to finance increased coverage of the *jatka* fisher rehabilitation programme and other management interventions, and pave the way for a more effective public-private partnership in the future (Bladon, 2016).

##### 3.7.3.4 Overall assessment

Based on this evidence, a feasible outer-bound undesirable counterfactual for hilsa could be the collapse of hilsa populations in Bangladesh within one to two decades, due to a combination of overfishing, climate change and environmental change. An alternative counterfactual, which is a feasible outer-bound desirable state, is where political will for institutional change, based on improved understanding of hilsa biology, effectively limits or reduces fishing pressure on hilsa populations, and mitigates some of the impacts of other anthropogenic activities, so that populations stabilise (Table 2).

### 3.8 Outstanding questions

Having developed our two feasible outer-bound counterfactuals, we highlight specific questions that would allow the most likely counterfactual to be established (Table 3). A key question is whether or not current management has the potential to protect hilsa populations. Due to the paucity of reliable time-series data, it is unclear whether the biological basis for current management rules is sound (Bladon, 2016). It is also yet to be seen whether *jatka* fisher compensation can incentivise compliance with the rules, and whether the DoF will increase coverage or improve targeting effectiveness of this compensation (Bladon, 2016).

**Table 3:** Outstanding areas of uncertainty relevant to establishing the projected counterfactual, and their associated management implications.

Driver	Uncertainty	Management implication
Institutional	• Will institutional capacity be sufficient to maintain compliance with management rules?	• Determines whether fishing regulations and conservation payments can have impact
	• Do management rules have a sound biological basis?	• Determines whether management focused on the protection of *jatka* can have impact
	• Will a regional hilsa fishery management plan be developed?	• Determines whether management could be undermined by activities of other countries
	• To what extent will the Conservation Trust Fund follow best practice?	• Will affect sustainability of conservation interventions

Social	• Will poverty decline in coastal areas?	• May influence fishing dependence, illegal fishing activities, and therefore the appropriateness of conservation interventions
	• Will *jatka* fisher compensation incentivise compliance?	• Influences potential for ecological impact

(Bio)economic	• Will industrialisation provide employment and reduce poverty?	• May influence fishing pressure, particularly on *jatka*, and thus appropriateness conservation interventions
	• How much will the industrial hilsa fishery expand?	• Determines the level of coast guard enforcement required
	• Is hilsa overexploited?	• Determines requirement for effort control
	• Is *jatka* overexploited?	• Determines appropriateness of management focus
	• Will industry trends have a significant impact on hilsa populations?	• Determines whether interventions should focus on protecting habitat or controlling fishing pressure

Physical	• Will climate change block migratory routes and affect feeding, spawning or migratory behaviour?	• Determines whether interventions should focus on protecting entire migratory route or just the spawning grounds that remain, and how adaptive interventions need to be
	• Will water pollution have a significant impact on hilsa populations and where?	• Determines whether fishery closures will provide effective protection
	• Will siltation and water diversion activities block migratory routes?	• Determines whether interventions should focus on protecting entire migratory route or just the spawning grounds that remain, and how adaptive interventions need to be

Further questions surround the extent of institutional change that might be introduced in the future. The need for regional transboundary management, human resource development, new financing mechanisms, improved monitoring and enforcement, community-based management, equality of fishing rights, habitat restoration and adaptive management are all recognised in the HFMAP (DoF, 2002), and although over a decade later most of these recommendations have not yet been implemented, areas in which progress has been made are financing and regional management. The creation of a CTF could be pivotal in terms of institutional change and sustainability of conservation interventions, but this will depend on the extent to which best practice is followed (Bladon, 2016; Bladon et al., 2014).

In terms of fishing pressure, although poverty decline could result in a decline in illegal fishing activities, it should be noted that the social and cultural importance of hilsa and of fishing as a livelihood activity may limit this. Moreover, it is unclear whether urban economic development and industrialisation will actually drive down poverty in remote coastal areas.

The potential impacts of climate change, polluting industries and water diversion activities are also uncertain, with implications for the appropriate placement of fishery closures, the appropriate focus of conservation interventions, and how adaptive they need to be.

### 3.9 Information needs

We now highlight key information requirements, in order to improve the robustness of the frame of reference as a tool for action and intervention evaluation (Table 4). There is a clear need to improve hilsa stock assessment by conducting fishery-independent surveys of biomass on an international regional scale, in order to help managers understand the proportion of hilsa that should be protected for sufficient spawning biomass and which life stages to target. If the current catch assessment survey continues to be used, more data on age structure and size composition are required, and the survey should be extended from major rivers and estuarine areas to cover the whole country, including marine areas. Any assessments should employ a standardised modelling framework so that they can support a regional management plan. Collection of commercial catch and effort data should be extended to the inland fishery, if possible, where pressure on hilsa populations and the distribution of fishing activities are unclear. A spatial analysis of fishing activities across all marine, riverine and estuarine areas would facilitate optimisation of fishery closures. More ecosystem research and modelling are needed to establish the effects of potential physical drivers of change, particularly climate change, pollution and water diversion activities. Finally, a rigorous assessment of the probable effectiveness of current management is required, in order to develop a dynamic baseline and counterfactual for future interventions. The hilsa literature is full of claims of positive impacts of fishery management interventions since 2007 (e.g. Rahman et al., 2012b), but none can attribute changes to specific interventions, due to the absence of baselines and counterfactuals.

**Table 4:** Key information needs for the hilsa fishery in Bangladesh.

• Regional stock assessment	There is a need for regional fishery-independent estimates of spawning stock biomass and juvenile recruitment, which could be used to support an international regional fishery management plan. Age structure and size composition of Bangladesh hilsa populations would also help to develop current fishery-dependent assessments.
• Improve catch and effort data collection	Catch assessment survey should be extended from the major rivers to cover the country more comprehensively. Currently commercial CPUE data is available only for the marine sector, but the inland sector provides about one third of estimated catch and so monitoring of these vessel numbers would shed light on the status of inland hilsa populations. Spatial analysis of actual fishing activities, as opposed to landings, would give a clearer picture of the fishery and could be used to optimise placement of fishery closures.
• Impact of climate change on hilsa populations	Existing reports of climate change impacts on hilsa populations are largely anecdotal. A clear link and mechanism for change must be established and the types of habitats that should be protected for increased resilience should be explored.
• Impact of water diversion activities on hilsa populations	Current reports of the impacts of damming on hilsa populations, though convincing, are still conjecture. Research linking quantitative habitat quality data to activities is needed.
• Impact of deforestation on hilsa populations	Given the role that mangroves tend to play in fish production, this gap in research should be addressed.
• Impact of pollution on hilsa populations	Quantitative studies of water quality, in relation to spawning and nursery areas, would help to ascertain whether pollution is undermining fishery closures.
• The potential of aquaculture	Advances in captive brood-stock development, breeding and grow-out of hilsa may help to supplement or reduce pressure on wild hilsa populations.
• Impact of fishery management, including the rehabilitation scheme	Currently there is limited evidence to attribute any changes to either the fishery closures or any element of the *jatka* fisher rehabilitation programme. Going forward, future rigorous impact evaluations will require the implementation of a long-term social and ecological monitoring programme.

## 4 Discussion

The task of developing successful fishery management interventions, and evaluating their contribution to sustainability, is always constrained by uncertainties arising from, for example, stochastic environmental variation or limited understanding of system dynamics – particularly resource user behaviour (Fulton et al., 2011; Nicholson and Possingham, 2007). Environmental modelling approaches to dealing with this uncertainty are available (Refsgaard et al., 2007), but they have received limited attention in the fisheries or conservation literature and are still rarely used in conservation decision-making (Milner-Gulland, 2012; Nicholson and Possingham, 2007). In fisheries, as in other fields of applied ecology, there is a widespread unwillingness among decision-makers to embrace uncertainty (Milner-Gulland and Shea, 2017; Walters, 2007). Managers have long expected scientists to provide single, clear predictions and management prescriptions from models parameterised with historical data, but the depth and complexity of understanding required for useful modelled predictions that reduce uncertainty to an acceptable level is often lacking, particularly in small-scale developing-world fisheries. Learning from the failures and successes of weather and climate science – particularly the dangers of failing to effectively communicate uncertainties to decision-makers and other stakeholders – alternative approaches to incorporating uncertainty into fisheries management decisions are surely required (Clark, 2001; Davies et al., 2015; Pidgeon and Fischhoff, 2011).

This study demonstrates how a useful frame of reference, comprising a baseline and counterfactual, can be developed to guide decision-making in complex, data-poor systems, even in the absence of reliable, predictable models. Similar in approach to scenario planning, this process can help to anticipate change, even when the projections themselves are qualitative and based on incomplete information (Clark, 2001). Although no quantitative counterfactual could be established for the hilsa fishery, the qualitative framework used here provides a way to break down and assess the drivers of potential change in a system – institutional, social, economic and physical – and their complex interactions, reducing the potential for unexpected outcomes (Larossa et al., 2016). The outer-bound feasible counterfactuals we developed may not be an accurate prediction of the future, but the process enabled critical evaluation of contradictions between studies and of the reliability of understanding and assumptions about trends in the hilsa fishery, and their likely causes. This allowed the identification of key areas of uncertainty, information needs and their implications for management. Thus, in addition to providing a frame of reference that can be used to evaluate potential future interventions, the study also provides a basis to consider the needs and opportunities for fishery improvements (Bladon, 2016). Moreover, the identification of outcomes that were likely in both counterfactual scenarios (e.g. climate change) highlighted areas that should be the focus of future research, if robust management interventions are to be designed and evaluated.

## 5 Conclusions

By implementing the frame of reference approach developed by Bull et al. (2015) in an entirely different social-ecological system, this study demonstrates the wider utility of the approach, beyond biodiversity offset policy-making. It is novel in its application of the frame of reference approach to fisheries, but it is also in line with new thinking that affords uncertainty greater importance in fisheries management planning, even where model-based predictions are unreliable or impossible (Davies et al., 2015). All fisheries are managed in the face of uncertainty, but management decisions should not be deferred by calls for further data collection or modelling when a reasonable frame of reference can be developed. Analysis of this kind is relatively rapid and inexpensive, and could thus be used on a regular basis to help guide an adaptive management approach to evaluation against a projected counterfactual (Bull, 2014). Finally, following this framework is one way to ensure that uncertainty is considered from the very beginning of an intervention design process (Refsgaard et al., 2007).
