# Supplementary material for: Developing a frame of reference for fisheries management and conservation interventions
Source: Fish Res. 2018 Dec;208:296–308. doi: 10.1016/j.fishres.2018.08.010 (PMC6179125; doi:10.1016/j.fishres.2018.08.010)
Supplement: Supplementary file 1 [file mmc1.docx]

**Selection of potential drivers of change (physical and social)**

# **Physical drivers**

## Climate change

Fish population variability is closely linked to environmental variability, and therefore to climate change (Klyashtorin and Lyubushin, 2007; Lehodey et al., 2006). Temperature, precipitation, and sea-level rise are thought to affect the spawning and migratory behaviour of hilsa, and ultimately production (Fernandes et al., 2015; Miah, 2015). Sea-level rise can be linked to coastal flooding and saline intrusion (Agrawala et al., 2003; Ali, 1999; Dasgupta, 2014; Miah, 2015). Precipitation is linked to river flow, run-off, and nutrient loadings – which affect primary productivity in coastal fisheries (Fernandes et al., 2015). Monsoon inundations are also linked to sedimentation in downstream channels , which can affect hilsa migratory routes (Ahsan et al., 2014; DoF, 2002).

Relevant data appropriate for analysis could only be found for temperature and precipitation. We obtained the data from the Bangladesh Meteorological Department (2014), collected from all five meteorological stations within the Meghna Estuary area of Bangladesh that is most well-known for hilsa (in Barisal, Bhola, Patuakhali, Chandpur, and Cox’s Bazar). Using these data, we calculated mean annual temperature and mean total annual rainfall for the period 1983 – 2014. We collected data for these years because catch monitoring of hilsa did not begin until 1983, and so this period is most useful for when looking for trends. We conducted statistical analyses using linear models (see Appendix B).

## Water diversion activities

The construction of dams and barrages for irrigation and flood control can reduce the input of freshwater and silt, reducing hilsa habitat availability and increasing salinity downstream (Gupta et al., 2012; Miao et al., 2010; Shibly and Takewaka, 2013). Dredging should open migratory routes for hilsa, whereas loop cutting, which results in the deposition of sediment, may have the opposite effect (Dewhurst-Richman et al., 2016).

## Forest cover

The importance of coastal forests, such as mangroves, as fish habitat – particularly nursery grounds – and their impacts on fishery yields are clear (Aburto-Oropeza et al., 2008; Hutchinson et al., 2014). No direct link between forest cover and hilsa is known, and hilsa do not appear to favour mangrove areas. However, it is probable that hilsa are affected by changes in mangrove forest cover through its effect on nutrient availability and primary productivity (Hutchinson et al., 2014). Mangroves also trap sediment and elevate land, reducing vulnerability to storm surges and saline intrusion inland (Auerbach et al., 2015). Forest cover in upstream zones has been linked to flooding, turbidity, and siltation (Bradshaw et al., 2007; van Dijk et al., 2009).

## Pollution

Water pollution is a clear environmental threat to marine and freshwater habitats in Bangladesh (BOBLME, 2011; Islam et al., 2017). Pollution is known to restrain phytoplankton growth and diversity, in turn reducing primary productivity (Huang et al., 2011), Petrochemicals can affect fish, their eggs and larvae and the plankton on which they feed, and can persist for long periods (Farrington, 2014). Pollution is considered a threat particularly to the inland hilsa fishery (BOBLME, 2012; Das, 2009; Islam, 2003), but marine petrochemical pollution has also anecdotally been linked to the availability of hilsa (Das, 2009).

# **Social drivers**

We obtained data online for population size and proportions in rural and urban areas (UN, 2015; 2018), and we obtained poverty trend data from the Bangladesh Poverty Assessment (World Bank, 2013).

Capture fisheries are not only essential for livelihood support but for direct consumption and dietary diversity, providing over 60 per cent of the animal protein in the Bangladeshi diet (Belton et al., 2014; FAO, 2014). Coastal communities in particular depend heavily on fisheries, including hilsa, for their livelihoods and food security (FRSS, 2013). Hilsa are sacred in Hindu mythology, feature in numerous ceremonial and religious festivals, and have been named the national fish of Bangladesh (Mohammed and Wahab, 2013; Sharma et al., 2012). Because supply does not meet demand, particularly during festival periods, market price is generally high (Padiyar et al., 2012) – although it was quite recently still one of the most widely consumed fish by all income groups (Belton et al., 2011). *Jatka* – being smaller and less tasty – has historically been the more affordable option for low income groups. These cultural values have bred a tradition of hilsa conservation – it is customary to buy a pair of hilsa on the day of *Vijay Dashami* (October) and not eat it again until *Basant Panchami* (February), a period which coincides roughly with the peak hilsa breeding season (Sharma et al., 2012). Nationally, the non-consumptive value of a well-managed hilsa fishery is estimated to be worth USD 167.5M – USD 355.7M (Mohammed et al. 2016).

# **References**

Agrawala, S., Ota, T., Ahmed, A.U., Smith, J., van Aalst, M., 2003. Development and climate change in Bangladesh: Focus on coastal flooding and the Sundarbans. Paris, Working Party on Global and Structural Policies, OECOD. Report number: COM/ENV/EPOC/DCD/DAC(2003)3/FINAL

Ahsan, D.A., Naser, M.N., Bhaumik, U., Hazra, S., Battacharya, S.B., 2014. Migration, spawning patterns and conservation of hilsa shad in Bangladesh and India. Academic Foundation, New Delhi. doi:10.1007/s13398-014-0173-7.2

Ali, A., 1999. Climate change impacts and adaptation assessment in Bangladesh. Clim. Res. 12, 109–116. doi:10.3354/cr012109

Auerbach, L.W., Goodbred, S.L., Mondal, D.R., Wilson, C.A., Ahmed, K.R., Roy, K., Steckler, M.S., Small, C., Gilligan, J.M., Ackerly, B.A. 2015. Flood risk of natural and embanked landscapes on the Ganges–Brahmaputra tidal delta plain. Nature Climate Change. 5, 153-157. doi: 10.1038/nclimate2472

[dataset] Bangladesh Meteorological Department, 2014. Bangladesh Meteorological Data 1983 - 2014. Bangladesh Meteorological Department, Dhaka.

Belton, B., van Asseldonk, I.J.M., Thilsted, S.H., 2014. Faltering fisheries and ascendant aquaculture: Implications for food and nutrition security in Bangladesh. Food Policy 44, 77–87. doi:10.1016/j.foodpol.2013.11.003

Belton, B., Karim, M., Thilsted, S., Murshed-E-Jahan, K., Collis, W., Phillips, M., 2011. Review of aquaculture & fish consumption in Bangladesh. Studies and Reviews 2011-53. WorldFish Center, Dhaka.

BOBLME, 2012. Management advisory for the Bay of Bengal hilsa fishery. Bay of Bengal Large Marine Ecosystem Regional Fisheries Management Advisory Committee, Phuket. http://www.boblme. org/BOBLME-2012-Leaflet-RFMAC_Advisory-hilsa/ (accessed: October 9th 2013).

BOBLME, 2011. Country report on pollution in BOBLME - Bangladesh. Bay of Bengal Large Marine Ecosystem Project, BOBLME-2014-Ecology-01.

Bradshaw, C.J.A., Sodhi, N.S., Peh, K.S.-H., Brook, B.W. 2007. Glob. Change Biol. 13, 2379-2395. doi: 10.1111/j.1365-2486.2007.01446.x

Das, M., 2009. Impact of commercial coastal fishing on the environment of Sundarbans for sustainable development. Asian Fish. Sci. 22, 157–167.

Dasgupta, S., 2014. Facing the hungry tide: Climate change, livelihood threats and household responses in coastal Bangladesh. Policy Research Working Paper 7148. World Bank Group, Washington, D.C.

Dewhurst-Richman, N., Mohammed, E.Y., Ali, M.L., Hassan, K., Wahab, M.A., Ahmed, Z.F., Islam, M.M., Bladon, A., Haldar, G.C., Ahmed, C.S., Majumder, M.K., Hossain, M.M., Rahman, A., Hussein, B. (2016)2016. Balancing Carrots and Sticks: Incentives for Sustainable Hilsa Fishery Management in Bangladesh. International Institute for Environment and Development, London.

van Dijk, A.I.J.M., van Noordwijk, M., Calder, I.R., Bruijnzeel, S.L.A., Schellekens, J. , Chappell, N.A. 2009. Forest–flood relation still tenuous – comment on ‘Global evidence that deforestation amplifies flood risk and severity in the developing world’ by C. J. A. Bradshaw, N.S. Sodi, K. S.-H. Peh and B. W. Brook. Glob. Change Biol. 15, 110–115. doi: 10.1111/j.1365-2486.2008.01708.x

DoF, 2002. Hilsa Fisheries Management Action Plan for Bangladesh. Department of Fisheries, Dhaka.

FAO, 2014. The state of world fisheries and aquaculture 2012. FAO, Rome. http://www.fao.org/docrep/016/i2727e/i2727e00.htm (accessed: October 4th 2012).

Farrington, J.W., 2014. Oil Pollution in the Marine Environment II: Fates and effects of oil spills. Environ. Sci. Policy Sustain. Dev. 56, 16–31. doi:10.1080/00139157.2014.922382

Fernandes, J.A., Susan, K., Hossain, M.A.R., Ahmed, M., Cheung, W.W.L., Lazar, A.N., Barange, M., 2015. Projecting marine fish production and catch potential in Bangladesh in the 21st century 3 under long-term environmental change and management scenarios. ICES J. Mar. Sci. 73, 1357–1369. doi:10.1093/icesjms/fsv217

FRSS, 2013. Fisheries Statistical Yearbook of Bangladesh 2011-2012. Fisheries Resources Survey System, Department of Fisheries, Dhaka.

Gupta, H., Kao, S.J., Dai, M., 2012. The role of mega dams in reducing sediment fluxes: A case study of large Asian rivers. J. Hydrol. 464, 447–458. doi:10.1016/j.jhydrol.2012.07.038

Huang, Y.J., Jiang, Z.B., Zeng, J.N., Chen, Q.Z., Zhao, Y., Liao, Y., Shou, L., Xu, X., 2011. The chronic effects of oil pollution on marine phytoplankton in a subtropical bay, China. Environ. Monit. Assess. 176, 517–530. doi:10.1007/s10661-010-1601-6

Hutchinson, J., Spalding, M., zu Emgassen, P., 2014. The role of mangroves in fisheries enhancement. The Nature Conservancy and Wetlands International, Cambridge.

Islam, M.A., Al-Mamun, A., Hossain, F., Quraishi, S.B., Naher, K., Khan, R., Das, S., Tamim, U., Hossain, S.M. and Nahid, F., 2017. Contamination and ecological risk assessment of trace elements in sediments of the rivers of Sundarban mangrove forest, Bangladesh. Marine Poll. Bull. 124, 356–366. doi:10.1016/j.marpolbul.2017.07.059

Islam, M.S., 2003. Perspectives of the coastal and marine fisheries of the Bay of Bengal, Bangladesh. Ocean Coast. Manag. 46, 763–796. doi:10.1016/S0964-5691(03)00064-4

Miah, M.S., 2015. Climatic and anthropogenic factors changing spawning pattern and production zone of Hilsa fishery in the Bay of Bengal. Weather Clim. Extrem. 1–7. doi:10.1016/j.wace.2015.01.001

Miao, W., Silva, S.D., Davy, B., 2010. Inland fisheries resource enhancement and conservation in Asia. RAP Publication 2010/22. FAO, Rome. http://www.fao.org/docrep/013/i1984e/i1984e00.htm (accessed: October 4th 2012).

Mohammed, E.Y., Ali, L., Ali, S., Hussein, B., Wahab, M.A., Sage, N. 2016. Hilsa’s non-consumptive value in Bangladesh: Estimating the non-consumptive value of the hilsa fishery in Bangladesh using the contingent valuation method, International Institute for Environment and Development, London

Mohammed, E.Y., Wahab, M.A., 2013. Direct economic incentives for sustainable fisheries management: The case of hilsa conservation in Bangladesh. IIED, London.

Padiyar, A., Belton, B., Swain, T., Momi, M.A., 2012. Hilsa market trends in Bangladesh and India. In: Anon (Ed.) Hilsa: Status of fishery and potential for aquaculture, proceedings of the regional workshop held in Dhaka, 16-17 September 2012. The WorldFish Bangladesh and South Asia Office, Dhaka, pp. 173-182.

Klyashtorin, L.B., Lyubushin, A.A., 2007. Cyclic Climate Changes and Fish Productivity. Moscow, VNIRO Publishing.

Lehodey, P., Alheit, J., Barange, M., Baumgartner, T., Beaugrand, G., Drinkwater, K.F., Fromentin, J.M., Hare, S.R., Ottersen, G., Perry, R.I., Roy, C., van der Lingen, C.D., Werner, F., 2006. Climate variability, fish and fisheries. J. Clim. 19, 5009–5030. doi:10.1175/JCLI3898.1

Sharma, A.P., Roy, N.C., Barman, B.C., 2012. Hilsa: Its social, cultural and religious importance. In: Anon (ed.) Hilsa: Status of fishery and potential for aquaculture, proceedings of the regional workshop held in Dhaka, 16-17 September 2012. The WorldFish Bangladesh and South Asia Office, Dhaka, pp. 216-223.

Shibly, A., Takewaka, S., 2013. Morphological changes and vegetation index variation along the western coastal zone of Bangladesh. In: Anon (Ed.) Proceedings of the 7th International Conference on Asian and Pacific Coasts (APAC 2013) Bali, Indonesia, September 24-26, 2013. http://repository.unhas.ac.id/bitstream/handle/123456789/7524/25.CP%2080.pdf?sequence=1 (accessed: March 3rd 2014).

[dataset] UN, 2015. UNdata. http://data.un.org/Default.aspx (accessed: January 14th 2015).

UN, 2018. World Urbanization Prospects, 2018 Revision. ST/ESA/SER.A/366. United Nations, New York.

World Bank, 2013. Bangladesh poverty assessment: assessing a decade of progress in reducing poverty 2000-2010. World Bank, Washington, D.C. http://www-wds.worldbank.org/external/default/WDSContentServer/WDSP/IB/2013/06/19/000333037_20130619115421/Rendered/PDF/785590NWP0Bang00Box0377348B0PUBLIC0.pdf (accessed: February 1st 2014).
