# Supplementary material for: Developing a frame of reference for fisheries management and conservation interventions
Source: Fish Res. 2018 Dec;208:296–308. doi: 10.1016/j.fishres.2018.08.010 (PMC6179125; doi:10.1016/j.fishres.2018.08.010)
Supplement: Supplementary file 2 [file mmc2.docx]

**Trends in drivers of change**

# **Trends in physical drivers**

## Climate

Over the period 1983 to 2014, mean monthly temperature peaked in May at 28.9°C ±0.1, with a low of 18.6°C ±0.2 in January (Fig. B.1a). Mean monthly total rainfall showed even more variation, from a low of 6.9 mm ±2.4 in January to a high of 922.3 mm ±43.0 in July (Fig. A.1b). This inter-annual variation is consistent with the main seasons; the dry winter season (December to February), pre-monsoon (March to May), monsoon (June to September) and post-monsoon (October to November) (Agrawala et al., 2003). Mean annual temperature in the study area has shown a significant upwards trend since 1983 (Fig. B.2a), whilst annual rainfall has declined significantly (Fig. B.2b). Both rainfall and temperature data were variable between stations, but trends in these data were consistent between stations. The local trends in temperature are consistent with regional studies; Bangladesh as a whole has experienced warming over the last 100 years (Adger et al., 2003; Agrawala et al., 2003). Regional precipitation trends are unavailable, but climate models tend to show an increase in precipitation during monsoon season – a trend which is consistent throughout South Asia (Agrawala et al., 2003; IPCC, 2013). Due to the dynamic morphology of the Ganges delta, no specific trend in regional sea-level rise could be found. However, approximately one fifth of Bangladesh lies within one metre of the high-water mark, making it particularly vulnerable to the effects of sea-level rise, which compound the enhanced storm surges associated with cyclones, increasing flood risk (Huq, 2001; Karim and Mimura, 2008).

**(a)**

**(b)**

**Figure B.1**: Inter-annual variation in **(a)** mean temperature and **(b)** mean total monthly rainfall from 1983-2014 across five meteorological stations in the study area. Error bars show 95% confidence intervals. Source: Bangladesh Meteorological Department (2014).

| **(a)** |  |
| --- | --- |
| **(b)** |  |

**Figure B.2**: **(a)** Mean annual temperature from five meteorological stations in southern Bangladesh, 1983-2014 (linear model: *r*^2^ = 0.169, p = 0.019); **(b)** Mean total annual rainfall from five meteorological stations in southern Bangladesh, 1983-2014 (linear model: *r*^2^ = 0.124, p = 0.047). Source: Bangladesh Meteorological Department (2014).

## Water diversion activities

The construction of dams and barrages for irrigation and flood control within and outside Bangladesh, together with estuary drainage and land reclamation projects, have led to hydrological and morphological changes in rivers (Ahsan et al., 2014; DoF, 2002). In particular, the construction of the Farakka Barrage on the Indian Ganges (Fig. A.3) has led to a decrease in dry season freshwater discharge in Bangladesh (Mirza, 1997), and branches and tributaries of the Padma and Brahmaputra – for example the Kumar River (Fig. B.3) – are reported to have dried up as a result (Ahsan et al., 2014). By reducing the input of freshwater and silt, damming has also probably increased salinity downstream (Gupta et al., 2012; Shibly and Takewaka, 2013). Some stretches of river are dredged to facilitate navigation and reduce flooding, and loop cutting – where the monsoon flood is diverted to eliminate meanders and loops, resulting in the deposition of large amounts of sediment – is a common practice in smaller rivers (Smith et al., 1998).

Although river channels are subject to constant changes in morphology through erosion and deposition, annual monsoon inundations deposit alluvial sediments from the Himalayas into the Bangladesh river system (Fig. B.3), where sandy islands form as they are carried into the Bay of Bengal (Ahsan et al., 2014; Curray and Moore, 1971; Shibly and Takewaka, 2013). High inter-annual and inter-study variability makes it difficult to draw conclusions on temporal trends in sediment loads entering the Bay (Islam et al., 1999).


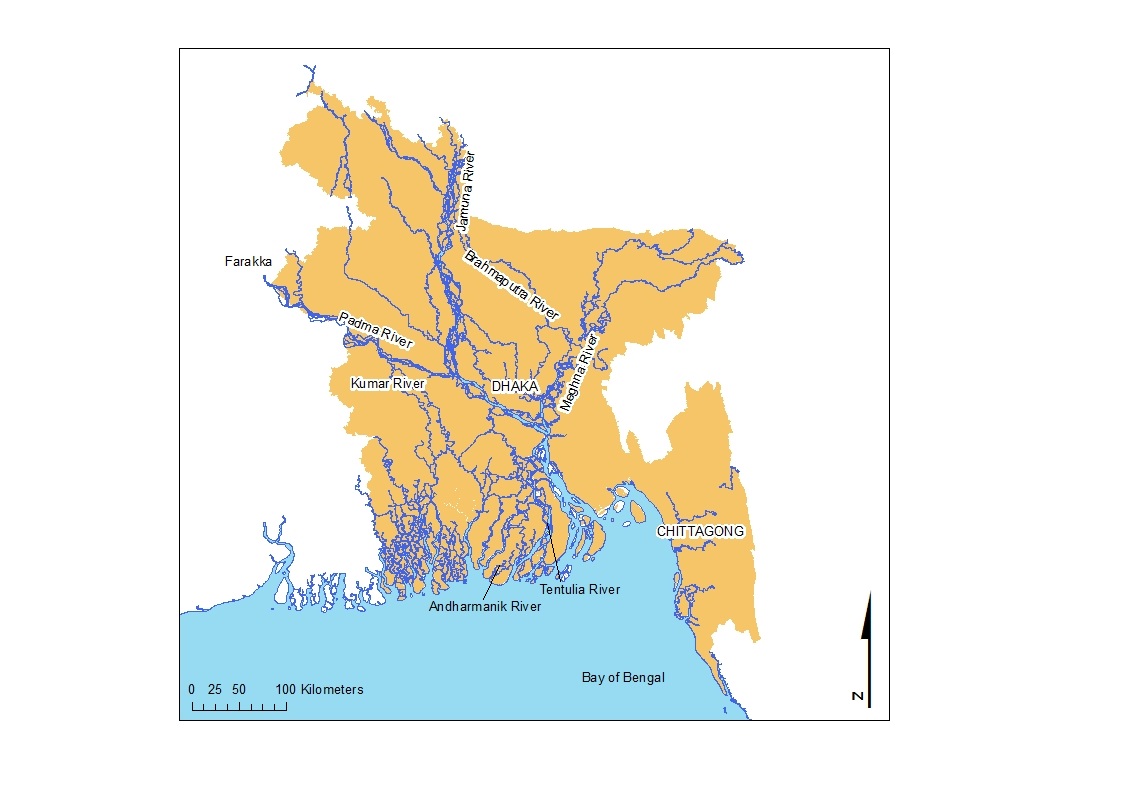
.

**Figure B.3**: Map showing the major rivers of the Ganges-Padma river system in Bangladesh (Ganges-Brahmaputra delta). Where the Ganges flows out of India, its main channel becomes the Padma River, which joins the Meghna River, which continues to flow into the Bay of Bengal. Farakka marks the approximate site of the Farakka Barrage, across the border in India. Capital letters indicate major cities.

## Forest cover

Forest cover is around 17 per cent in Bangladesh. Although this is an estimated 10 per cent of its original extent, recent net annual change appears to be stable or even positive (BBS, 2012; Laurance, 2007). Much of this cover is moist deciduous forest, but the south-western coastal area is dominated by the Sundarbans mangroves and the freshwater swamp forests that lie behind them. Studies on forest cover have focused on this western coastal zone due to the important role mangroves play in shoreline stabilisation, storm protection, flood control and fishery support (Iftekhar and Islam, 2004). Mangrove loss has been caused by timber collection and by increased salinity resulting from unregulated encroachment of shrimp farming, storm surges, and reduced freshwater flow (Hoq, 2007; Iftekhar and Islam, 2004).

## Pollution

With few and poorly enforced environmental standards, industrial effluents and untreated municipal waste and sewage are released from urban areas into the river systems of Bangladesh, particularly around Dhaka (see Fig. B.3), eventually reaching the Bay of Bengal (BBS, 2009; Hoque and Clarke, 2013; Karn and Harada, 2001). Principal sources of inland pollution are factories, including garment factories (Karn and Harada, 2001). Water quality in polluted rivers has declined since 2000 (BOBLME, 2011), and there is evidence for a rapid increase in the production of hazardous industrial waste in Bangladesh between 1994 and 2007 (Waste Concern, 2010). Eutrophication caused by the expansion of intensive agriculture and aquaculture activities is also an increasing problem (BOBLME, 2011; Islam, 2003). In coastal waters, petrochemical pollution is produced by mechanised vessels, shipbuilding, and ship breaking activities (Chowdhury et al., 2017; Hossain et al., 2016). Most research on marine pollution in Bangladesh has been focused on the Sundarban mangrove system and Chittagong (see Fig. B.3), a large port and ship breaking area (BOBLME, 2011; Islam et al., 2017; Kumar et al., 2016).

# **Trends in social drivers**

The population of Bangladesh has been growing since before independence, and it has one of the highest population densities in the world (UN, 2015). Despite this growth, there has been a steady decline in poverty over the last decade or so and two thirds of the population are now above the poverty threshold (UN, 2015; World Bank, 2013). The Government’s vision for 2021 is to bring the numbers below the poverty threshold down to less than 15 per cent (GED, 2012). The proportion of people living in urban areas increased from 10 per cent in 1971 to 36 per cent in 2018 and, since independence, rural-urban migration has accounted for two thirds of this urban growth (Afsar, 2003; UN, 2018). Extreme poverty is most prevalent in rural areas and poverty declines have been much more substantial in the west of Bangladesh than the east, where the hilsa fisheries are concentrated (World Bank, 2013).

The DoF estimates that fisheries support the livelihoods of 11 per cent of the total population (FRSS, 2013) and fishers are often described as the poorest and most vulnerable social group in the country; they are usually landless with little education, low income, and few or no other livelihood opportunities (Deb and Haque, 2011; Islam, 2012; Leterme et al., 2004). Fishing bans have been implemented in hilsa nursery areas, and affected households are compensated with rice, but malnutrition is a risk even for those households who receive compensation (Islam et al., 2016).

# **Trends in economic drivers**

## Industrialisation

The economy of Bangladesh is rapidly developing, largely through industrialisation and exports. It had an average national GDP growth rate of six per cent over the decade to 2012 (BBS, 2012) and is classified as one of the ‘Next Eleven’ – a group of countries recognised for their potentially large, fast-growing markets (Goldman Sachs, 2007). More than half of GDP is currently generated by the service sector, followed by industry (30 per cent), and agriculture, forestry, and fisheries (16 per cent; BBS, 2013). The garment industry, which has emerged over the last two or three decades, provides the most foreign exchange earnings (80 per cent; CIA, 2014). Other growing industries are shipbuilding and ship breaking; the world’s largest ship-breaking area is in the Bangladesh city of Chittagong (Hossain et al., 2016). Over 45 per cent of the population is employed in the agricultural sector, but although there has been a substantial increase in food grain production in recent years due to modernisation and mechanisation, agriculture is failing to absorb the rising labour force and its contribution to GDP is projected to fall (BBS, 2012; GED, 2012). Bangladesh has substantial untapped oil and gas reserves and is promoting international exploration (BBS, 2012). Although it has limited coal reserves, there are also plans to increase coal-fired power generation (Allchin, 2015).

## Fishery sector

Official production statistics show a general upward trend in total fish production since 1983 (Table B.1).

**Table B.1:** Fishery production (metric tonnes) in Bangladesh from 1983-84 to 2011-12 (DoF, 2014a)

| **Year** | **Inland capture** | **Marine capture** | **Total capture** | **Aquaculture** | **Country Total** | **Inland hilsa** | **Marine hilsa** | **Total hilsa** |
| --- | --- | --- | --- | --- | --- | --- | --- | --- |
| 1983-84 | 471595 | 164882 | 636477 | 117025 | 753502 | 90082 | 56000 | 146082 |
| 1984-85 | 462605 | 187563 | 650168 | 123811 | 773979 | 73328 | 71050 | 144438 |
| 1985-86 | 441799 | 207401 | 649200 | 144723 | 793923 | 94794 | 96294 | 191091 |
| 1986-87 | 431006 | 217579 | 648585 | 166100 | 814685 | 91167 | 103814 | 194981 |
| 1987-88 | 423598 | 227582 | 651180 | 175925 | 827105 | 78551 | 104950 | 183501 |
| 1988-89 | 424140 | 233281 | 657421 | 183505 | 840926 | 81641 | 110311 | 191952 |
| 1989-90 | 423872 | 239063 | 662935 | 192592 | 855527 | 112408 | 113943 | 226351 |
| 1990-91 | 443404 | 241538 | 684942 | 210993 | 895935 | 66809 | 115358 | 182167 |
| 1991-92 | 479742 | 245474 | 725216 | 226863 | 952079 | 68356 | 120106 | 188462 |
| 1992-93 | 532419 | 250492 | 782911 | 237743 | 1020654 | 74715 | 123115 | 197830 |
| 1993-94 | 573376 | 253044 | 826420 | 264190 | 1090610 | 71370 | 121161 | 192531 |
| 1994-95 | 591145 | 264650 | 855795 | 317073 | 1172868 | 84420 | 129115 | 213535 |
| 1995-96 | 609151 | 269702 | 878853 | 379087 | 1257940 | 80625 | 126660 | 207285 |
| 1996-97 | 599900 | 274704 | 874604 | 485864 | 1360468 | 83230 | 131204 | 214434 |
| 1997-98 | 615949 | 272818 | 888767 | 574812 | 1463579 | 81634 | 124105 | 205739 |
| 1998-99 | 649418 | 309797 | 959215 | 593202 | 1552417 | 73809 | 140710 | 214519 |
| 1999-00 | 670465 | 333799 | 1004264 | 657120 | 1661384 | 79165 | 140367 | 219532 |
| 2000-01 | 688920 | 379497 | 1068417 | 712640 | 1781057 | 75060 | 154654 | 229714 |
| 2001-02 | 688435 | 415420 | 1103855 | 786604 | 1890459 | 68250 | 152343 | 220593 |
| 2002-03 | 709333 | 431908 | 1141241 | 856956 | 1998179 | 62944 | 136088 | 199032 |
| 2003-04 | 732067 | 455207 | 1187274 | 914752 | 2102026 | 71001 | 184838 | 255839 |
| 2004-05 | 859269 | 474597 | 1333866 | 882091 | 2215957 | 77499 | 198363 | 275862 |
| 2005-06 | 956686 | 479810 | 1436496 | 892049 | 2328545 | 78273 | 198850 | 277123 |
| 2006-07 | 1006761 | 487438 | 1494199 | 945812 | 2440011 | 82445 | 196744 | 279189 |
| 2007-08 | 1060181 | 497573 | 1557754 | 1005542 | 2563296 | 89900 | 200100 | 290000 |
| 2008-09 | 1123925 | 514644 | 1638569 | 1062801 | 2701370 | 95970 | 202951 | 298921 |
| 2009-10 | 1029937 | 517282 | 1547219 | 1351979 | 2899198 | 115179 | 198574 | 313753 |
| 2010-11 | 1054585 | 546333 | 1054585 | 1460769 | 3061687 | 114520 | 225325 | 339845 |
| 2011-12 | 957095 | 578620 | 1139388 | 1726067 | 3261782 | 114475 | 232037 | 346512 |

Aquaculture production has increased steadily to contribute over 50 per cent in 2012, whereas capture fishery production has declined since 2008. In 2012, inland capture fisheries contributed about 30 per cent and marine capture fisheries 18 per cent.

## Hilsa fishery

There are large and highly variable numbers of fishing vessels in use, mostly unregistered: an estimated 100,000 vessels inland and 25,000 in the marine fishery (Rahman et al., 2010). Estimated numbers of hilsa fishers in Bangladesh range from 300,000 to 500,000 (Haldar, 2004; Islam et al., 2016; Rahman et al., 2014).

Artisanal fishing practices are strongly influenced by traditional knowledge of lunar periodicity and tides passed down from generation to generation (Sharma et al., 2012). Inland stocks are exploited using traditional non-mechanised boats (*chandi, khosa* and *dingi*), and some mechanised boats. According to the literature, the gears most commonly used are set gill nets (*chandi* *jal*) and drift gill nets (*gulti, kona* and *current jal*), with variable mesh sizes, but numerous others are in use (e.g. clasp nets, set bag nets and barrier nets; Rahman et al., 2012b). *Jatka* are targeted during their downstream migration, particularly at night when large numbers can be intercepted using illegal monofilament *current* *jal*, mosquito seine nets (*moshari jal*), set bag nets (*behundi jal*) and fixed encircling nets (*char ghera jal*; Rahman et al., 2010; Siddique, 2009). About 60 per cent of marine landings can be attributed to mechanised and non-mechanised boats and gill nets, and the rest to the industrial sub-sector that uses small trawlers to fish up to 250 km from the coast (Haldar, 2004). Artisanal fishers may fish from their own boat or a boat owned by a *mahajon*^[[1]](#footnote-1)^ (Alam et al., 2012). They often fish in groups of 4-20 to a boat, each fisher receiving small shares of the catch (Mome and Arnason, 2007), whereas fishers working for industrial trawler companies can earn fixed salaries and catch-based bonuses (Kleih et al., 2003).

Traditionally, professional fishers in coastal areas were ‘low caste’ Hindus and fishing was considered taboo for Muslims, but now there are increasing numbers of poor and landless Muslims engaging in fishing, and wealthy Muslims investing in the fishing business (Hussain and Hoq, 2010). Artisanal fishers often take loans for gear or boats from moneylenders and commission agents, which commit the fishers to sell back to or through these middlemen exclusively and for lower than market price (Alam et al., 2012; Islam et al., 2016). Juvenile hilsa (*jatka*) are targeted during their downstream migration, particularly at night when large numbers can be intercepted. Illegal use of a monofilament *current* *jal* net is reported to have increased in recent years (Islam et al., 2016). Violence and piracy is widespread in the marine and inland fisheries (Kleih et al., 2003); pirates, known as *dacoits*, often have political connections and increase in numbers during peak hilsa season (R. Mohkles, Centre for Natural Resource Management, personal communication, 23^rd^ May 2014).

The hilsa marketing system requires icing and transportation, and so it is long and complex. 2-2.5 million workers are estimated to be involved in the market chain and ancillary activities such as boat-building, including women and children (Ahmed, 2007; Mohammed and Wahab, 2013). Fishers rarely sell catch directly to consumers, but instead operate through a number of intermediaries or middlemen (Alam, 2010; Rahman et al., 2013). Alam et al. (2012) identified four to six main intermediaries in the hilsa supply chain, depending on the type of market. The marketing system varies from place to place, as do the local names of the intermediaries, but its basic three-level structure is described in Fig. B.4. The key players are the aratdars, who connect buyers and sellers of fish by handling the auctioning process at wholesale markets, taking a commission of up to five per cent. They often invest in fishing activities by giving loans to fishers, wholesalers/suppliers (known locally as paikars or beparis) and retailers. They usually have their own storage facilities (arat) and may employ staff. There are two types of aratdar: those who collect fish from local paikars, mobile collectors (farias), mohajons, or directly from fishers, and sell in local markets; and those who operate second auctions in urban areas or more distant markets. Paikars/beparis can play the role of local suppliers at the primary market level or as wholesalers supplying fish from local and distant wholesale markets to retailers, who then sell on to consumers at fish markets and as street vendors. Only LC (letter of credit) paikars are licensed to export hilsa overseas.


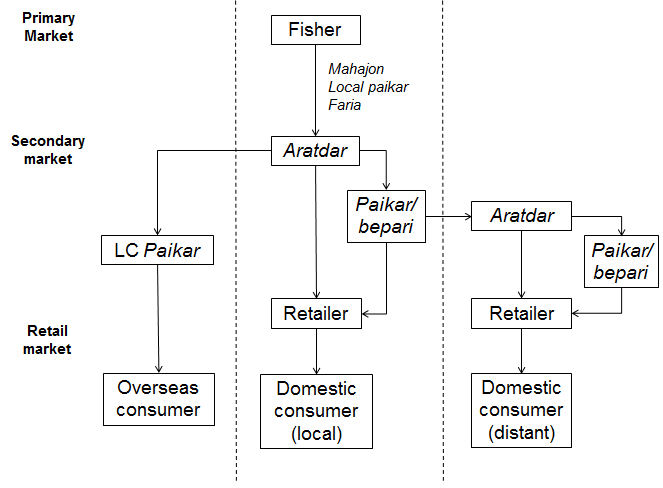


**Figure B.4**: A simplified representation of the domestic and foreign market chains for hilsa in Bangladesh (Alam et al., 2012). Three market levels exist. LC = letter credit. The terms *paikar, bepari, faria* have different meanings in different regions.

These intermediaries have complete control of the marketing system; fishers lack market information and bargaining power, particularly in remote areas with poor transport links, so they have no choice but to sell to intermediaries (Haque, 2011; Islam et al., 2016; Islam, 2003). Furthermore, the presence of so many players in the supply chain raises the retail price and the costs to the fishers, locking them into cycles of debt which traditional microfinance schemes have largely failed to drag them out of (Alam, 2012; Ali et al., 2010; Uraguchi and Mohammed, 2016). Supply chain analysis conducted on hilsa caught in the coastal Patuakhali district indicate that marketing profits received by intermediaries are relatively high, with only 55 per cent of retail price going to the fishers (Ahmed, 2007). Another analysis conducted in Chandpur district found fishers receiving 31 per cent of retail price, with the major share of profit going to the mahajons (Alam et al., 2012). Dadandars (aratdars and mahajons) in particular play a key role in determining market prices, through the extension of credit (dadan) which requires fishers to sell their catch back to/through them below the market price (Kleih et al., 2003). Although the dadan is interest free, this price differential is an informal equivalent of interest.

Block ice is used for preservation on trawlers and mechanised artisanal boats which fish for more than one day (Kleih et al., 2003). If the transportation time from primary market to retail market is in excess of six hours, it is usually iced (Ahmed, 2007). Alam et al. (2012) found post-harvest loss to be minimal, except in peak fishing season when supply exceeds the availability of ice. Hilsa are preferred fresh, but dry and wet salting and salt-fermentation are used as methods of long-term preservation, particularly during peak fishing season (Alam, 2010). It cannot be sun-dried due to its high lipid content. Low value hilsa may be frozen and sold more cheaply on the domestic market. High value hilsa for the export market (Table B.2) are frozen whole using semi-IFQ (individual quick freezing), sometimes in shrimp processing plants (Alam et al., 2012).

**Table B.2**: Officially reported hilsa export tonnage (metric tonnes) and earnings since 2002 (DoF, 2014b). Unknown figures left blank.

| Year | Hilsa export (mt) | Per cent of total hilsa catch exported | Export earning (million BDT) | Export earning (million USD) |
| --- | --- | --- | --- | --- |
| 2002-03 | 1148 | 0.6 | 150.00 | 1.91 |
| 2003-04 | 1930 | 0.8 | 790.00 | 10.07 |
| 2004-05 | 3584 | 1.3 | 519.50 | 6.62 |
| 2005-06 | 3672 | 1.3 | 696.10 | 8.87 |
| 2006-07 | 3433 | 1.3 | 648.10 | 8.26 |
| 2007-08 | 2647 | 0.9 | 754.50 | 9.62 |
| 2008-09 | 3680 | 1.2 | 1490.60 | 19.00 |
| 2009-10 | 3107 | 1.0 | 1241.20 | 15.82 |
| 2010-11 | 8539 | 2.5 | 3524.90 | 44.93 |
| 2011-12 | 6174 | 1.8 | 2940.00 | 37.48 |
| 2012-13 | 523^*^ | - | 243.70 | 3.11 |
| 2013-14 | 0 | 0 | 0.00 | 0.00 |

^*^ Ban implemented half way through year

**Table B.3**: Boats operated per year and average catch (metric tonnes) per boat per year in the marine sector in Bangladesh from 1983-84 to 2005-06 (Mome and Arnason, 2007).

| **Year** | **Number of boats** | | | **Average catch/boat/year (mt)** | | |
| --- | --- | --- | --- | --- | --- | --- |
|  | **Mechanised** | **Non-mechanised** | **Total** | **Mechanised** | **Non-mechanised** | **Total** |
| 1983-84 | 3347 | - | 3347 | 16.70 | - | 16.70 |
| 1984-85 | 3000 | - | 3000 | 23.70 | - | 23.70 |
| 1985-86 | 2887 | 3802 | 6682 | 30.60 | 2.08 | 14.40 |
| 1986-87 | 2887 | 3800 | 6680 | 32.90 | 2.36 | 15.50 |
| 1987-88 | 2882 | 3509 | 6389 | 31.90 | 3.76 | 16.40 |
| 1988-89 | 2880 | 3509 | 6389 | 33.00 | 4.37 | 17.30 |
| 1989-90 | 2880 | 3509 | 6389 | 33.10 | 5.32 | 17.80 |
| 1990-91 | 2880 | 3509 | 6389 | 33.90 | 5.06 | 18.10 |
| 1991-92 | 2880 | 3509 | 6389 | 35.40 | 3.14 | 18.80 |
| 1992-93 | 2880 | 3509 | 6389 | 36.50 | 5.13 | 19.30 |
| 1993-94 | 2880 | 3509 | 6389 | 36.10 | 4.94 | 19.00 |
| 1994-95 | 2880 | 3509 | 6389 | 38.70 | 5.03 | 20.20 |
| 1995-96 | 2880 | 3509 | 6389 | 37.90 | 4.95 | 19.80 |
| 1996-97 | 2880 | 3509 | 6389 | 39.90 | 4.64 | 20.50 |
| 1997-98 | 2880 | 3509 | 6389 | 38.30 | 3.72 | 1.40 |
| 1998-99 | 2880 | 3509 | 6389 | 42.30 | 5.35 | 22.00 |
| 1999-00 | 18982 | 7177 | 26169 | 6.30 | 2.94 | 5.40 |
| 2000-01 | 18982 | 6377 | 25369 | 6.91 | 3.67 | 6.00 |
| 2001-02 | 18982 | 6377 | 25369 | 6.93 | 3.24 | 5.90 |
| 2002-03 | 18982 | 6377 | 25369 | 6.02 | 3.42 | 5.36 |
| 2003-04 | 18982 | 6377 | 25369 | 8.30 | 4.28 | 7.29 |
| 2004-05 | 18982 | 6377 | 25369 | 9.00 | 4.33 | 7.82 |
| 2005-06 | 18982 | 6377 | 25369 | 9.00 | 4.48 | 7.84 |

# **Trends in institutional drivers**

## Administrative hierarchy of Bangladesh

The institutional arrangement of fisheries management in Bangladesh is hierarchical (Fig. B.5). Bangladesh comprises eight administrative divisions, which are in turn subdivided into sixty-four districts. Each district comprises sub-districts called *upazilas*, which are further divided into unions, and finally wards, which contain a number of villages. District and division level administration is led by central Government officials. Urban local governance operates through six city corporations (Dhaka, Chittagong, Rajshahi, Khulna, Barisal, and Sylhet) each led by a Mayor. Unions and *upazilas* in rural areas are governed by democratically elected local councils, called *parishads* (Fig. B.5)*.*


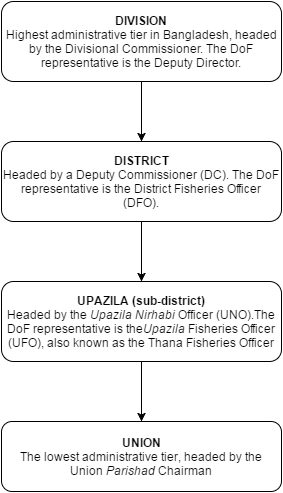


Figure B.5: Administrative hierarchy in Bangladesh. DoF = Department of Fisheries.

Union *parishads* are made up of representatives from each village and a chairman. *Upazila parishads* include union *parishad* chairmen, mayors, an elected chairman and two vice chairmen. Central Government bureaucrats also have influence at the *upazila* level via the *upazila nirbahi* officers and ministry representatives, and central Government bureaucracy is reported to impede local administration (Ahsan, 2010). Local village leaders tend to make community decisions.

The DoF, within the Ministry of Fisheries and Livestock (MoFL), is the principal organisation for the management and development of fish resources, represented at divisional, district, and sub-district levels. DoF activities are supported by autonomous organisations under the administrative control of MoFL: the Bangladesh Fisheries Development Corporation (BFDC) was established to promote the fishing industry and develop landing and marketing facilities; and the Bangladesh Fisheries Research Institution (BFRI) conducts research. Both the DoF and BFRI has been criticised for a lack of human resources, and BFRI for a lack of coordination between research bodies (DoF, 2002; Islam et al., 2016).

## National fishery regulation and management

In 1983, the Department of Fisheries enacted the Marine Fisheries Ordinance^[[2]](#footnote-2)^ and Rules^[[3]](#footnote-3)^ (MoFL, 1983) under which waters less than 40 m in depth at high tide are reserved for the artisanal fishery and waters beyond this point are for the industrial fishery – although trawlers continue to fish up to 20 m depth (Ali et al., 2010; Islam, 2003). All coastal and inland waters are managed under the Protection and Conservation of Fish rules, 1985, in accordance with the Fish Protection and Conservation Act, 1950 (MoFL, 1985). Monitoring and enforcement of these rules by the DoF is supported by the Navy, coast guard, police, Rapid Action Battalion, Air Force, Border Guard and local administrations.

In 1986 the New Fisheries Management Policy emphasised tackling the overexploitation of resources and the inequality of fishing rights (Islam et al., 2016). Then in 1998 a National Fisheries Policy (NFP) was prepared with the objectives of increasing fish production and promoting economic growth through sustainable fisheries management and aquaculture in inland open water and the sea, while conserving biodiversity and ecological function and alleviating poverty (MoFL, 1998). It did not, however, formulate a specific strategy for the management of the artisanal hilsa fishery.

All mechanised and, since 2001, non-mechanised vessels in the marine fishery must pay registration fees when commissioned, as well as annual vessel and fishing license fees (Islam et al., 2016). There is a restriction on the number of trawlers fishing in marine waters, but no effort restriction has been imposed on the artisanal fisheries, and although the river fisheries have been through various systems of short-term licensing, they are now classified as ‘open access’ (Ali et al., 2010; Rab, 2009). These ‘open-access’ river fisheries are, however, under complex systems of customary or traditional rights held by investors, which are not well documented (Dastidar, 2009)

## Hilsa fishery management

### *Protection of spawning hilsa*

Spawning hilsa are protected with a ban on hilsa fishing throughout the country for 15 days of the perceived peak breeding season, with the aim of minimising disturbance to spawning and recruitment (Bladon, 2016). Monitoring for compliance with this ban is targeted within a 7000 km^2^ area that is thought to cover important spawning grounds. But inconsistencies in ban periods between years and locations indicate a lack of communication between scientists and authorities (Islam et al., 2016).

### *Implementation of Protection and Conservation of Fish Act and Rules, 1950*

Under the Protection and Conservation of Fish Act and Rules, 1950^[[4]](#footnote-4)^ (MoFL, 1985), special operations for *jatka* conservation ban all activities related to *jatka* (catching, transportation, marketing, selling and possession) between 1st November and 31st July across the country. *Jatka* was originally defined as a hilsa fish of less than 23 cm long, but this has recently been amended to 25 cm (Islam et al., 2016). Use of monofilament gillnets (*current jal*) under 4.5 cm mesh size was banned in 1988, and both the use and production^[[5]](#footnote-5)^ of those under 10 cm mesh size is now banned, although not strictly enforced (Islam et al., 2016). The Fish Rules are implemented by the DoF’s *Upazila* Fishery Officers (UFOs), and more recently the Navy and Coast Guard in the main rivers, which are reported to have improved enforcement (DoF, 2002). It is not currently financially or logistically possible to enforce rules nationwide, so enforcement is targeted to the 152 *upazilas* where *jatka* are distributed, and particularly those areas that are thought to be important nursery grounds. However, the Fish Act has numerous institutional weaknesses, and there have been instances of social and political interference with implementation (Islam et al., 2016). For instance, the Mobile Court Ordinance, 2007, allows a magistrate to operate a mobile court to deal with offenses on site, but resources are still lacking to gather mobile courts in time to do so.

### *Jatka fisher rehabilitation*

In recognition of the socioeconomic hardships imposed by the fishing rules, in 2004 the DoF introduced the *jatka* fisher rehabilitation programme, which aims to improve the socioeconomic condition of affected fishers living inside and around sanctuary areas and thereby to incentivise compliance with the fishing bans (Islam et al., 2016). This is largely based on the distribution of compensation in the form of rice during ban periods (centered on the sanctuary fishing bans), which is funded through the pre-existing national Vulnerable Group Feeding (VGF) programme (Ahmed et al., 2009; Uraguchi, 2011). Allocations and coverage increased from 10 kg per household for one to three months for 145,335 households in 2008, to 40 kg per household for four months (February to May, period of peak *jatka* abundance) for 224,102 households in 2014 (Table B.4). However, the extent to which the food compensation actually incentivises compliance with regulations is probably limited by distributional issues and undermined by poor enforcement (Bladon et al., 2016; Haldar and Ali, 2014; Islam et al., 2016).

In 2008, the rehabilitation programme was extended to provide a smaller proportion of households with alternative livelihood support such as rickshaws, vans, livestock and grants for small businesses. This support might be a more appropriate incentive than food distribution but, despite calls for increased coverage, the numbers of households receiving alternative livelihood support have actually decreased in recent years (Table B.4), probably due to a lack of resources and needs assessment (Bladon, 2016; Bladon et al., 2016; Islam et al., 2016; Siddique, 2009).

Since 2003, awareness has been raised on the importance and status of the fishery, particularly *jatka*, and on the Fish Act and Rules, through boat rallies, mass media, and distribution of leaflets and posters (Bladon et al., 2016).

Table B.4: Distribution of rice compensation and alternative livelihood support (DoF, 2014c). *Upazila* = subdistrict.

| RICE COMPENSATION | | | | | ALTERNATIVE LIVELIHOOD SUPPORT | | | |
| --- | --- | --- | --- | --- | --- | --- | --- | --- |
| Year | **No. of *upazilas* (and districts)** | **Total volume distributed (mt)** | **No. of households** | **Monthly allocation household (kg)** | **No. of *upazilas* (and districts)** | **Total allocated amount (USD)** | **No. of households** | **Amount allocated per household (USD)** |
| 2004-2005 | - | 1,000.00 | - | - | - | - | - | - |
| 2005-2006^*^ | - | - | - | - | - | - | - | - |
| 2006-2007 | - | 1,546.00 | - | - | - | - | - | - |
| 2007-2008 | 59 (10) | 4,360.00 | 145,335 | 10 | 20 (4) | - | - | - |
| 2008-2009 | 59 (10) | 5,730.08 | 143,252 | 10 | 20 (4) | - | - | - |
| 2009-2010 | 59 (10) | 19,768.60 | 164,740 | 30 | 20 (4) | 17,157.08 | 4,388 | 3.91 |
| 2010-2011 | 85 (15) | 14,470.64 | 186,264 | 20 | 20 (4) | 45,816.23 | 6,869 | 6.67 |
| 2011-2012 | 85 (15) | 22,351.68 | 186,264 | 30 | 20 (4) | 58,854.60 | 7,785 | 7.56 |
| 2012-2013 | 88 (16) | 24,747.48 | 206,229 | 30 | 20 (4) | 2,928.24 | 1,743 | 1.68 |
| 2013-2014 | 88 (15) | 36,296.32 | 224,102 | 40 | 28 (6) | 1,759.15 | 1,165 | 1.51 |

^*^ Rice not provided

# **References**

Adger, N., Huq, S., Brown, K., Conway, D., Hulme, M., 2003. Adaptation to climate change in the developing world. Prog. Dev. Stud. 3, 179–195. doi:10.1191/1464993403ps060oa

Aburto-Oropeza, O., Ezcurra, E., Danemann, G., Valdez, V., Murray, J., Sala, E., 2008. Mangroves in the Gulf of California increase fishery yields. Proc. Natl. Acad. Sci. U. S. A. 105, 10456–10459. doi:10.1073/pnas.0804601105

Afsar, R., 2003. Internal migration and the development nexus: the case of Bangladesh. Regional Conference on Migration, Development and Pro-Poor Policy Choices in Asia, 22–24 June 2003 in Dhaka, Bangladesh. doi:10.1057/dev.2009.89

Agrawala, S., Ota, T., Ahmed, A.U., Smith, J., van Aalst, M., 2003. Development and climate change in Bangladesh: Focus on coastal flooding and the Sundarbans. Paris, Working Party on Global and Structural Policies, OECOD. Report number: COM/ENV/EPOC/DCD/DAC(2003)3/FINAL

Ahmed, N., 2007. Value chain analysis for hilsa marketing in coastal Bangladesh. Aquac. News 33, 14–20.

Alam, A.K.M.N., 2010. Post-harvest loss reduction in fisheries in Bangladesh: A way forward to food security. Final Report #PR 5/08. Bangladesh Agricultural University. http://www.nfpcsp.org/agridrupal/sites/default/files/Nowsad_Alam-PR5-08.pdf (accessed: February 1st 2013).

Alam, M.S., 2012. Hilsa Fisheries Management in Bangladesh: A paradigm in natural resources conservation, in: Hilsa: Status of Fishery and Potential for Aquaculture, proceedings of the regional workshop held in Dhaka, 16-17 September 2012. Dhaka, WorldFish Centre, pp. 224-338.

Alam, F., Palash, S., Mian, I.A., Day, M.M., 2012. Marketing of major fish species in Bangladesh: a value chain analysis. Rome, FAO.

Allchin, J., 2015. Low-lying Bangladesh targets jump in coal use. <https://www.ft.com/content/241059fa-9424-11e5-bd82-c1fb87bef7af> (accessed: October 27^th^ 2017).

Ali, M.L., Hossain, M.B., Rokunuzzaman, M., Bhadra, S., 2010. Access to fisheries resources by the poor fishers for income generation and livelihood and their coping strategies during lean and ban fishing period in Bangladesh. Final Report CF # 9/08. Bangladesh Centre for Advanced Studies, Dhaka. http://www.nfpcsp.org/agridrupal/sites/default/files/CF-9_0f_08_Liaquat_Ali.pdf (accessed: May 11th 2013).

Ahsan, D.A., Naser, M.N., Bhaumik, U., Hazra, S., Battacharya, S.B., 2014. Migration, spawning patterns and conservation of hilsa shad in Bangladesh and India. Academic Foundation, New Delhi. doi:10.1007/s13398-014-0173-7.2

[dataset] Bangladesh Meteorological Department, 2014. Bangladesh Meteorological Data 1983 - 2014. Bangladesh Meteorological Department, Dhaka.

BBS, 2009. Statistical Pocket Book of Bangladesh 2008. Bangladesh Bureau of Statistics, Government of Bangladesh, Dhaka.

BBS, 2012. Statistical Yearbook. Bangladesh Bureau of Statistics, Ministry of Planning, Dhaka.

BBS, 2013. GDP 2013-2014. Bangladesh Bureau of Statistics, Ministry of Planning, Dhaka.

BOBLME, 2011. Assessments of the Indian mackerel (*Rastrelliger kanagurta*) and the Hilsa shad (*Tenualosa ilisha*) fisheries in the BOBLME countries. Bay of Bengal Large Marine Ecosystem Project, BOBLME-2011-Ecology-09.

Chowdhury, K.M.A, Mili, M.I.J., Akhter, S., Ahmed, K.A., 2017. Pollution by shipping industry in the northern Bay of Bengal: A review study. IJIRM, 3.

CIA, 2014. The World Factbook 2014. Central Intelligence Agency. https://www.cia.gov/library/publications/download/ (accessed: December 2nd 2015).

Curray, J.R., Moore, D.D., 1971. Growth of Bengal Deep Sea Fan and Denundation in the Himalayas, in: Whitakeo, J.H.M. (Ed.), Submarine Canyons and Deep Sea Fans. Dowden, Hutchinson & Ross, Stroudsburg, pp. 236–245.

Dastidar, R., 2009. Capitalist development and technological innovation in open-water fisheries: Impacts on traditional 'water-slave' fishing communities of Southeastern Bangladesh. PhD thesis. National University of Singapore.

Deb, A.K., Haque, C.E., 2011. “Sufferings Start from the Mothers’ Womb”: Vulnerabilities and livelihood war of the small-scale fishers of Bangladesh. Sustainability 3, 2500–2527. doi:10.3390/su3122500

DoF, 2002. Hilsa Fisheries Management Action Plan for Bangladesh. Department of Fisheries, Dhaka.

[dataset] DoF, 2014a. Bangladesh Fisheries Landings Data 1983-2013. Department of Fisheries, Dhaka.

[dataset] DoF, 2014b. Bangladesh hilsa export data. Department of Fisheries, Dhaka.

[dataset] DoF, 2014c. Food compensation and alternative income generating support. Department of Fisheries, Dhaka.

FRSS, 2013. Fisheries Statistical Yearbook of Bangladesh 2011-2012. Fisheries Resources Survey System, Department of Fisheries, Dhaka.

GED, 2012. Perspective Plan of Bangladesh 2010-2021. General Economics Division, Planning Commission, Dhaka.

Goldman Sachs, 2007. The N-11: More than an acronym. Global Economics Paper No: 153. Goldman Sachs. <http://www.onuncuplan.gov.tr/oik1/Belgeler/Altyap%C4%B1%20Dok%C3%BCmanlar%C4%B1-%20Projeksiyon%20%C3%87al%C4%B1%C5%9Fmalar%C4%B1/2007-Goldman%20Scachs-Global%20Economics%20Paper%20No.153.pdf> (accessed: October 27^th^ 2017).

Gupta, H., Kao, S.J., Dai, M., 2012. The role of mega dams in reducing sediment fluxes: A case study of large Asian rivers. J. Hydrol. 464, 447–458. doi:10.1016/j.jhydrol.2012.07.038

Haldar, G.C., 2004. Present status of the hilsa fisheries in bangladesh: a report on hilsa management and conservation studies conducted under the ARDMCS, GEF component, FFP. Chandpur.

Haldar G.C. and Ali L., 2014. The Cost of Compensation: Transaction and Administration Costs of Hilsa Management in Bangladesh. IIED Working Paper, IIED; London.

Haque, A., 2011. Fish-market chain and fishers’ incomes in Sherpur district. In: Fox, J (Ed.) Rural livelihoods and protected landscapes: co-management in the wetlands and forests of Bangladesh. Nishorgo Network, Bangladesh, pp. 33–46.

Hoq, M.E., 2007. An analysis of fisheries exploitation and management practices in Sundarbans mangrove ecosystem, Bangladesh. Ocean Coast. Manag. 50, 411–427. doi:10.1016/j.ocecoaman.2006.11.001

Hoque, A., Clarke, A., 2013. Greening of industries in Bangladesh: Pollution prevention practices. J. Clean. Prod. 51, 47–56. doi:10.1016/j.jclepro.2012.09.008

Hossain, M.S., Fakhruddin, A.N.M., Chowdhury, M.A.Z., Gan, S.H., 2016. Impact of ship-Breaking activities on the coastal environment of Bangladesh and a management system for its sustainability. Environ. Sci. Policy 60, 84–94.

Hussain, M.G., Hoq, M., 2010. Sustainable management of fisheries resources of the Bay of Bengal: Compilation of national and regional workshop reports. Support to Sustainable Management of the BOBLME Project, Bangladesh Fisheries Research Institute, Dhaka. SBOBLMEP Pub./Rep. 2. 122 p.

Huq, S., 2001. Climate change and Bangladesh. Science 294, 1617. doi:10.1126/science.294.5547.1617

Iftekhar, M.S., Islam, M.R., 2004. Degeneration of Bangladesh’s Sundarbans mangroves: a management issue. Int. Forest. Rev. 6, 123–135. doi:10.1505/ifor.6.2.123.38390

IPCC, 2013. Technical Summary. In: Stocker, T.F., Qin, D., Plattner, G. K., et al. (Eds.) Climate Change 2013: The Physical Science Basis. Contribution of Working Group I to the Fifth Assessment Report of the Intergovernmental Panel on Climate Change, Cambridge University Press, Cambridge and New York.

Islam, M., 2012. Poverty in small-scale fishing communities in Bangladesh: Contexts and responses. PhD thesis. University of Bremen.

Islam, M., Mohammed, E.Y., Ali, L., 2016. Economic incentives for sustainable hilsa fishing in Bangladesh: An analysis of the legal and institutional framework. Mar. Policy 68, 8–22.

Islam, M.A., Al-Mamun, A., Hossain, F., Quraishi, S.B., Naher, K., Khan, R., Das, S., Tamim, U., Hossain, S.M. and Nahid, F., 2017. Contamination and ecological risk assessment of trace elements in sediments of the rivers of Sundarban mangrove forest, Bangladesh. Marine Poll. Bull. 124, 356–366. doi:10.1016/j.marpolbul.2017.07.059

Islam, M.R., Begum, S.F., Yamaguchi, Y., Ogawa, K., 1999. The Ganges and Brahmaputra rivers in Bangladesh: Basin denudation and sedimentation. Hydrol. Processes. 13, 2907-2923.

Islam, M.S., 2003. Perspectives of the coastal and marine fisheries of the Bay of Bengal, Bangladesh. Ocean Coast. Manag. 46, 763–796. doi:10.1016/S0964-5691(03)00064-4

Karim, M.F., Mimura, N., 2008. Impacts of climate change and sea-level rise on cyclonic storm surge floods in Bangladesh. Glob. Environ. Chang. 18, 490–500. doi:10.1016/j.gloenvcha.2008.05.002

Karn, S.K., Harada, H., 2001. Surface water pollution in three urban territories of Nepal, India, and Bangladesh. Environ. Manage. 28, 483–496. doi:10.1007/s002670010238

Kleih, U., Alam, K., Dastidar, R., Dutta, U., Oudwater, N., Ward, A., 2003. Livelihoods in coastal fishing communities, and the marine fish marketing system of Bangladesh: synthesis of participatory rural appraisals in six villages, and assessment of the marketing system. NRI Report No. 2712. Natural Resources Institute, Greenwich University, London.

Kumar, A., Ramanathan, A., Prasad, M.B.K., Datta, D., Kumar, M., Sappal, S.M., 2016. Distribution, enrichment, and potential toxicity of trace metals in the surface sediments of Sundarban mangrove ecosystem, Bangladesh: a baseline study before Sundarban oil spill of December, 2014. Environ. Sci. Pollut. Res. 23, 8985. doi:10.1007/s11356-016-6086-6

Laurance, W.F., 2007. Forest destruction in tropical Asia. Science. 93, 1544–1550.

Leterme, J.L., Haldar, G.C., Kumar, P., 2004. Baseline socioeconomic survey and impact assessment of hilsa fishing ban on the socioeconomic condition of fishermen. Report No-38.13. Aquatic Resources Development, Management and Conservation Studies, GEF Component, Fourth Fisheries Project, Department of Fisheries, Dhaka.

Mirza, M.M.Q., 1997. Hydrological changes in the Ganges system in Bangladesh in the post-Farakka period. Hydrol. Sci. J. 42, 613–631. doi:10.1080/02626669709492062

MoFL, 1998. National Fisheries Policy. Ministry of Fisheries and Livestock, Dhaka.

MoFL, 1985. Protection and Conservation of Fish Rules. Ministry of Fisheries and Livestock, Dhaka.

MoFL, 1983. Marine Fisheries Rules 1983. Ministry of Fisheries and Livestock, Dhaka.

Mohammed, E.Y., Wahab, M.A., 2013. Direct economic incentives for sustainable fisheries management: The case of hilsa conservation in Bangladesh. IIED, London.

Mome, M.A., Arnason, R., 2007. The potential of the artisanal hilsa fishery in Bangladesh: an economically efficient fisheries policy. Final project, The United Nations University. <http://www.unuftp.is/static/fellows/document/masud07prf.pdf> (accessed: October 27^th^ 2017).

Rab, M.A., 2009. River fisheries management in Bangladesh: Drawing lessons from Community Based Fisheries Management (CBFM) experiences. Ocean Coast. Manag. 52, 533–538. doi:10.1016/j.ocecoaman.2009.08.001

Rahman, M.A., Emran, M., Islam, S., 2010. Status of hilsa fisheries in Bangladesh. Regional Consultation on Preparation of Management Plan for Hilsa Fisheries. Chittagong, Bangladesh, 7 - 8 February 2010. BOBP-IGO/ RC-HF2/5. Bay of Bengal Programme Intergovernmental Organisation, Chittagong, Bangladesh.

Rahman, Alam, M.A., Hasan, S.J., Zaher, M., 2012. Hilsa (*Tenualosa ilisha*) fishery management in Bangladesh. In: Anon (Ed.) Hilsa: Status of fishery and potential for aquaculture, proceedings of the regional workshop held in Dhaka, 16-17 September 2012. The WorldFish Bangladesh and South Asia Office, Dhaka, pp. 40-60.

Rahman, M., Khatun, S., Hossain, M.B., Hassan, M.N., Nowsad, A.A.K.M., 2013. Present scenario of landing and distribution of fish in Bangladesh. Pakistan J. Biol. Sci. 16, 1488–1495.

Rahman, H.Z., Wahab, M.A., Choudhury, L.A., 2014. Hilsa and hilsa fishermen: Exploring conservation-livelihood win-wins. Power and Participation Research Centre (PPRC), Dhaka, Bangladesh.

Sharma, A.P., Roy, N.C., Barman, B.C., 2012. Hilsa: Its social, cultural and religious importance. In: Anon (ed.) Hilsa: Status of fishery and potential for aquaculture, proceedings of the regional workshop held in Dhaka, 16-17 September 2012. The WorldFish Bangladesh and South Asia Office, Dhaka, pp. 216-223.

Shibly, A., Takewaka, S., 2013. Morphological changes and vegetation index variation along the western coastal zone of Bangladesh. In: Anon (Ed.) Proceedings of the 7th International Conference on Asian and Pacific Coasts (APAC 2013) Bali, Indonesia, September 24-26, 2013. http://repository.unhas.ac.id/bitstream/handle/123456789/7524/25.CP%2080.pdf?sequence=1 (accessed: March 3rd 2014).

Siddique, M.A.L., 2009. Conservation of juvenile hilsa (jatka) in Bangladesh: Need to address the livelihood of fishers. In: Challenges for Diadromous Fishes in a Dynamic Global Environment. American Fisheries Society Symposium 69. American Fisheries Society, Halifax, pp. 757-768.

Smith, B.D., Haque, A.K.M.A., Hossain, M.S., Khan, A., 1998. River dolphins in Bangladesh: Conservation and the effects of water development. Environ. Manage. 22, 323–335. doi:10.1007/s002679900108

[dataset] UN, 2015. UNdata. http://data.un.org/Default.aspx (accessed: January 14th 2015).

UN, 2018. World Urbanization Prospects, 2018 Revision. ST/ESA/SER.A/366. United Nations, New York.

Uraguchi, Z.B., Mohammed, E.Y., 2016. Harnessing market forces for financial inclusivity in marine and coastal conservation: Lessons from market systems development approach. In: Mackelworth, P. (Ed.), Marine Transboundary Conservation and Protected Areas. Earthscan, Oxford.

Waste Concern, 2010. Hazardous waste management in Bangladesh: a country inventory. Department of Environment, Ministry of Environment and Forests, Dhaka, Bangladesh.

World Bank, 2013. Bangladesh poverty assessment: assessing a decade of progress in reducing poverty 2000-2010. World Bank, Washington, D.C. http://www-wds.worldbank.org/external/default/WDSContentServer/WDSP/IB/2013/06/19/000333037_20130619115421/Rendered/PDF/785590NWP0Bang00Box0377348B0PUBLIC0.pdf (accessed: February 1st 2014).

Wright, J., Hill, N., Roe, D., Rowcliffe, M., Kumpel, N., Day, M., Booker, F., Milner-Gulland, E.J., 2016. Reframing the concept of ‘alternative livelihoods’. Cons. Biol. 30, 7-13. doi:10.1111/cobi.12607

1. Relatively wealthy and powerful ‘armchair fishers’, who do not fish, but retain a large proportion of the catch or profit. Like other moneylenders, having given loans or credit in the form of boats or nets, they have complete control over fishing activities and market prices. [↑](#footnote-ref-1)
2. An ordinance is a law passed by a local administration. [↑](#footnote-ref-2)
3. Rules define the guidelines that must be followed for the successful implementation of an act, which is a law passed by Government. [↑](#footnote-ref-3)
4. Amended in in 1985 by the Protection and Conservation Ordinance, 1982. [↑](#footnote-ref-4)
5. Use was banned in 1988 but the 2002 ban on production, marketing, importation, and possession was blocked by producers until resolution by the High Court in 2005. [↑](#footnote-ref-5)
