# Supplementary material for: Developing a frame of reference for fisheries management and conservation interventions
Source: Fish Res. 2018 Dec;208:296–308. doi: 10.1016/j.fishres.2018.08.010 (PMC6179125; doi:10.1016/j.fishres.2018.08.010)
Supplement: Supplementary file 3 [file mmc3.docx]

**Trends in hilsa**

# **Movement**

Hilsa are typically understood to be anadromous (migrating from marine to freshwater to spawn), but movements are complex and varied and there may be some permanent riverine and marine populations in Bangladesh (Bhaumik, 2015; Blaber et al., 2003; Rahman et al., 2012a). *Jatka* can be found in all major rivers in Bangladesh, but major nursery grounds have been identified by experimental fishing in the lower reaches and estuary of the Meghna River and on the coast from Kuakata to Dubla Char (Fig. 3; Haldar, 2004). Within the Bay of Bengal, otolith microchemistry and allozyme variation provide evidence of substantial gene flow between groups of hilsa, indicating that fish in Bangladesh may also have spawned in India or Myanmar, and vice versa (Milton and Chenery, 2001; Salini et al., 2004). Movements are strongly influenced by environmental conditions: water salinity, turbidity, temperature, pH, dissolved O^2^, and phytoplankton availability – indicated by chlorophyll concentration or nutrient levels (Table C.1). Water quality maps, generated using acceptable ranges of water quality parameters for hilsa, have been combined with catch data to explore its migration route through the Ganges-Padma river system (Ahsan et al., 2014). Upstream migration is associated with the increased flow and turbidity that comes with the monsoon, and fluctuations in hilsa abundance in different areas have been linked to monsoon intensity (BOBLME, 2011). The peak migration period is therefore generally understood to start somewhere between May and July, and to continue to October or November, although a shorter migration season in winter months from January to March has also been observed (Ahsan et al., 2014; Islam et al., 2016). Catch data from Bangladesh indicate that hilsa prefer water of at least 20 metres’ depth for migration (Ahsan et al., 2014; Blaber et al., 2003; Rahman et al., 2012a).

There are reports of large areas of hilsa spawning habitat having been lost in the upper region of the country due to water diversion activities, particularly in the upper Padma and Kumar rivers (Blaber et al., 2003). Absence of hilsa has been reported in some rivers, and migratory distances are now estimated to reach only 50-100 km, probably due to the disturbance of migratory routes and sedimentation forming sandy islands (DoF, 2002; Miah, 2015; Rahman et al., 2010; Sharma, 2012). Pollution is reportedly making other areas of river unsuitable for hilsa – particularly the Buriganga, which receives effluent from Dhaka (S.N. Chowdhury, Winrock International, personal communication, 13th May 2014), and the Andharmanik (Bladon, 2016; Hasan et al., 2015; Miah, 2015). There are also anecdotal reports of reduced availability of hilsa in coastal areas due to petrochemical pollution (Das 2009).

**Table C.1**: Historical hilsa fecundity data from 1968-2007 (Haldar, 2004; Milton and Chenery, 2001).

| **Date** | **Habitat** | **Length (cm)** | **Weight (g)** | **Egg numbers** |
| --- | --- | --- | --- | --- |
| 1968 | Padma-Meghna | 22.5-48.3 | - | 900,000-2,000,000 |
| 1977 | Meghna | 38.0-52.0 | - | 382,702-1,821,420 |
| 1982 | Padma-Meghna | 33.0-51.0 | - | 600,000-1,500,000 |
| 1992 | Padma (Goalunda) | 26.6-51.1 | 228-1635 | 179,000-1,302,000 |
| 1998 | Meghna | 28.7-52.3 | - | 226,000-1,931,000 |
| 2001 | Bangladesh | 17.1-41.5 | - | 108,500-1,993,846 |
| 2002-2004 | Ramgoti (Laxmipur) | 35.5-47.0 | 448-1300 | 135,600-1,703,200 |
| 2002-2004 | Kuakata (Patuakhali) | 26.8-46.2 | 220-1270 | 209,000-1,088,200 |
| 2006-2007 | Chandpur/Ramgoti | 24.0-48.0 | 220-1130 | 112,554-950,625 |

# **Reproduction**

Fecundity varies with body size and habitat (Rahman et al., 2012a; Table C.1), and although views on temporal trends are inconsistent (Blaber et al., 2003; Haldar, 2004; Miah, 2015), historical data suggests an overall decrease in fecundity in the last four decades (Table C.1). Spawning (like migration) is influenced by exogenous factors (Table C.1). Spawning occurs year round, with a peak in September and October following the monsoon flooding (Ahsan et al., 2014; Bhaumik, 2015; Hasan et al., 2016; Rahman et al., 2012a).

# **Growth**

From the point when the larvae become fry, hilsa feed on plankton (mainly phytoplankton) and so their growth is heavily influenced by phytoplankton availability, which can be estimated by measuring chlorophyll concentration (Table C.2; Hasan et al., 2015). Growth parameters have been estimated based on length-frequency data collected between 1992 and 2009 (Table C.3). Although these data are difficult to compare, because only one study adjusted data for gillnet selectivity (Rahman and Cowx, 2008), they have been validated by other studies using length-at-age data based on otolith microchemistry (Blaber et al., 2003; Milton and Chenery, 2001; Rahman and Cowx, 2008). A decrease in size at first capture can be seen between 1992 and 2000, indicating that many hilsa were being caught before reaching maturity (Haldar, 2004), but it appears to have increased again since, coinciding with the introduction of more fisheries management measures (Table C.3).

**Table C.2**: Threshold values of physical and chemical parameters for hilsa spawning and nursery activities in Bangladesh (Ahsan et al., 2014).

|  | Spawning activities | Nursery activities |
| --- | --- | --- |
| Depth | ≥ 20 m for migration and pre-spawning congregation | Comparatively shallower depth |
| Turbidity (NTU) | 100-140 | 70-80 |
| Temperature (°C) | 29.3-30.2 | 29.8-30.8 |
| Salinity (ppt) | < 0.1 | < 0.1 |
| Dissolved O_2_ (ppm) | 5.0-6.8 | 4.8-6.8 |
| pH | 7.70-8.30 | 7.9-8.40 |
| Chlorophyll (µg/l) | 0.114-0.180 | 0.140-0.180 |

# **Mortality**

Total mortality comprises natural mortality (loss of stock through natural causes) and fishing mortality (removal of stock through fishing). Estimates of mortality parameters have been made from length-frequency data collected over time, though again it should be noted that methodologies differed between studies, making comparison difficult (Table C.3). Estimated instantaneous rates of natural mortality are variable, ranging from a low of 0.98 in 1998 to highs of 1.36 in 2002 and 2009. Instantaneous rates of total mortality also peaked in 2002 at 3.51, compared with a low of 2.34 in 1998. Instantaneous rates of fishing mortality increased overall from 1992 to 2009, peaking in 1999 (2.49), and well exceeded natural mortality in every year. Estimates declined from 2002 to 2009, coinciding with the introduction of new fisheries management measures. Fishing mortality was higher for inland samples than marine samples.

Hilsa landings data also provide some indication of trends in fishing mortality. Annual hilsa landings have increased since 1983 (Fig. 2). Hilsa are harvested throughout the year from the Padma River, the Meghna Estuary and the inshore waters of the Bay of Bengal (FRSS, 2013). Despite an upward trend in total landings, marine landings have actually increased at a much faster rate than inland landings, which have stayed fairly stable. However, marine CPUE appears to have declined.

# **Stock status**

Stock assessments have been conducted in Bangladesh with hilsa population parameters estimated using length-frequency data (Amin et al., 2008, 2004, 2002; BOBLME, 2010). Although this approach is not rigorous, it provides some of the only available indicators of hilsa abundance in Bangladesh and results are quite consistent. Between 1992 and 2009, estimated exploitation rate increased overall and fishing mortality rate was consistently higher than natural mortality, although since 1999 there has been a downward trend in exploitation and fishing mortality rate (Table C.3). These studies broadly concluded that the fishery was overexploited, and attributed this to growth and recruitment overfishing, although some reports made contradictory recommendations for exploitation levels to be increased (DoF, 2002; Rahman et al., 2012a, 2012b). The only study that analysed marine and inland samples separately concluded that both populations were still under the maximum acceptable effort limit (Rahman and Cowx, 2008). While inland hilsa stocks were found to be slightly overexploited, Rahman & Cowx (2008) concluded that the biologically optimal yield could still be obtained at a higher exploitation level for marine populations.

The Bay of Bengal Large Marine Ecosystem (BOBLME) Project used a productivity-susceptibility analysis (PSA) to assess hilsa stocks in the Bay of Bengal – a risk assessment approach with fewer data requirements than stock assessment, where ‘productivity’ is a composite measure of several key parameters (fecundity, catch rates, growth rates, age composition, mortality index and probability of breeding) and ‘susceptibility’ of attributes which determine susceptibility to threats (including protected areas, range and habitat quality; BOBLME, 2010). The study identified a declining trend in most of the productivity parameters in hilsa, but concluded that although there is evidence of recruitment overfishing in Bangladesh, stocks are not depleted or in need of rebuilding.

Systems dynamics simulation modelling has also been conducted using hilsa population parameters from the studies above (Bala et al., 2014). The study predicted a Maximum Sustainable Yield (MSY) of 268,000 tonnes (much lower than landings estimates in that time frame) and found growth rates of *jatka* and spawning adults to be very small, concluding that stocks are under ‘severe stress’ and vulnerable to overfishing. However, it did not show a decline in productivity; simulated weights of standing stock increased from 290,000 tonnes in 2004 to 380,000 tonnes in 2014 under current harvesting practice. These weights are higher than other estimates of 218,000 tonnes in 2003 (Mome and Arnason, 2007) and an average of 95,144 tonnes from 1997-1999 (Amin et al., 2004).

Periodic experimental CPUE surveys have been conducted in the Meghna river, and some authors noted a slight decline between 1998 and 2011 (BOBLME, 2010; Rahman et al., 2012b). Yet these surveys shed no light on the status of marine populations, nor are they directly comparable. More recently, Sharma (2012) used time-series marine catch and effort data to estimate overall biomass with dynamic surplus production models and determined stock to be 15-30 per cent below optimal yield targets – ‘marginally overfished to overfished’. Mome & Arnason (2007), on the other hand, conducted a bioeconomic assessment indicating that current estimates of marine effort were 33 per cent higher than the level of effort for MSY (Mome and Arnason, 2007). Both of these studies used official catch and effort data that are known to be unreliable.

**Table C.3**: Growth, mortality, and exploitation parameters for the hilsa fishery in Bangladesh. Only in 1998**^**^** were samples from marine and inland waters analysed separately and adjusted for gillnet selectivity. ‘?’ indicates unclear trend. Adapted from Sharma (2012)^*^ and Rahman & Cowx (2008)^**^. Mortalities are instantaneous rates and thus can exceed a value of one.

|  | **1992^*^** | **1995^*^** | **1996^*^** | **1997^*^** | **1998^*^** | **1998^**^** | | | **1999^*^** | **2000^*^** | **2002^*^** | **2003^*^** | **2009^*^** | **Overall trend** |
| --- | --- | --- | --- | --- | --- | --- | --- | --- | --- | --- | --- | --- | --- | --- |
|  |  |  |  |  |  | **Inland** | **Marine** | **Mean** |  |  |  |  |  |  |
| **Asymptotic length (*l*_∞_ in cm)** | 61.10 | 58.30 | 60.00 | 61.50 | 66.00 | 58.80 | 61.00 | 59.90 | 60.00 | 62.50 | 53.70 | 54.60 | 53.00 | -? |
| **Growth constant (*k*)** | 0.74 | 0.74 | 0.99 | 0.83 | 0.67 | 0.82 | 0.80 | 0.81 | 0.82 | 0.72 | 0.86 | 0.67 | 0.83 | +? |
| **Growth performance index** | - | 3.40 | 3.55 | 3.46 | 3.46 | 3.45 | 3.47 | 3.46 | 3.47 | 3.45 | 3.40 | 3.30 | 3.37 | ? |
| **Length at first capture (l_c_ in cm)** | 35.0 | 30.0 | 30.3 | 29.81 | 27.06 | - | - | - | 22.80 | 13.12 | 19.87 | 21.21 | 26.00 | - |
| **Total mortality (*Z*)** | 2.41 | 2.61 | 3.19 | 3.29 | 3.43 | 2.38 | 2.30 | 2.34 | 3.77 | 2.79 | 3.51 | 3.07 | 3.23 | +? |
| **Natural mortality (*M*)** | 1.16 | 1.18 | 1.41 | 1.28 | 1.25 | 1.00 | 0.98 | 0.99 | 1.28 | 1.17 | 1.36 | 1.15 | 1.36 | + |
| **Fishing mortality (*F*)** | 1.25 | 1.43 | 1.78 | 2.01 | 2.18 | 1.38 | 1.32 | 1.35 | 2.49 | 1.62 | 2.16 | 1.92 | 1.87 | + |
| **Exploitation rate (*E*)** | 0.52 | 0.55 | 0.56 | 0.61 | 0.63 | 0.58 | 0.57 | 0.58 | 0.66 | 0.58 | 0.61 | 0.62 | 0.58 | + |
| **Maximum yield per recruit (*E*_max_)** | - | - | 0.71 | 0.69 | 0.60 | 0.61 | 0.65 | 0.63 | 0.59 | 0.46 | 0.58 | 0.63 | 0.57 | - |

# **References**

Ahsan, D.A., Naser, M.N., Bhaumik, U., Hazra, S., Battacharya, S.B., 2014. Migration, spawning patterns and conservation of hilsa shad in Bangladesh and India. Academic Foundation, New Delhi*.* doi:10.1007/s13398-014-0173-7.2.

Amin, S.M.N., Rahman, M.A., Haldar, G.C., Mazid, M.A., Milton, D., 2002. Population dynamics and stock assessment of hilsa shad, *Tenualosa ilisha*, in Bangladesh. Asian Fish. Sci. 15, 123-128.

Amin, S.M.N., Rahman, M.A., Haldar, G.C., Mazid, M.A., 2004. Stock assessment and management of *Tenualosa ilisha* in Bangladesh. Asian Fish. Sci. 17, 51–59.

Amin, S.M.N., Rahman, M.A., Haldar, G.C., Mazid, M.A., Milton, D.A., 2008. Catch per unit effort, exploitation level and production of hilsa shad in Bangladesh. Asian Fish. Sci. 21, 175–187.

Bhaumik, U., 2015. Migration of hilsa shad in the Indo-Pacific region – A review. Int. J. Curr. Res. Acad. Rev. 3, 139-155.

Bala, B.K., Arshad, F.M., Alias, E.F., Sidique, S.F., Noh, K.M., Rowshon, M.K., Islam, Q.M.M., Islam, M.M., 2014. Sustainable exploitation of hilsa fish (*Tenualosa ilisha*) population in Bangladesh: Modeling and policy implications. Ecol. Model. 283, 19–30. doi:10.1016/j.ecolmodel.2014.03.013

Blaber, S.J.M., Milton, D., Chenery, S.R., 2003. New insights into the life history of Tenualosa ilisha and fishery implications. In: Limburg K.E. and Waldman J.R. (eds.) Biodiversity, status and conservation of the world's shads: American Fisheries Society Symposium 35, Baltimore, 20-23 May 2001. American Fisheries Society, Bethseda, pp. 223-240.

Bladon, A.J., 2016. Conservation payments in data-poor developing-world fisheries. PhD thesis. Imperial College London.

BOBLME, 2010. Status of the hilsa (*Tenualosa ilisha*) fishery management in the Bay of Bengal. Bay of Bengal Large Marine Ecosystem Project, BOBLME-2010-Ecology-01.

Das, M., 2009. Impact of commercial coastal fishing on the environment of Sundarbans for sustainable development. Asian Fish. Sci. 22, 157–167.

FRSS, 2013. Fisheries Statistical Yearbook of Bangladesh 2011-2012. Fisheries Resources Survey System, Department of Fisheries, Dhaka.

Haldar, G.C., 2004. Present status of the hilsa fisheries in bangladesh: a report on hilsa management and conservation studies conducted under the ARDMCS, GEF component, FFP. Chandpur.

Hasan, K., Wahab, A., Ahmed, Z.F., Mohammed, E.Y., 2016. Food and feeding ecology hilsa (*Tenualosa ilisha*) in Bangladesh’s Meghna river basin. IIED Working Paper. IIED, London. <http://pubs.iied.org/16609IIED/> (accessed: October 27^th^ 2017).

Hasan, K., Wahab, A., Ahmed, Z.F., Mohammed, E.Y., 2015. The biophysical assessments of the hilsa fish (*Tenualosa ilisha*) habitat in the lower Meghna, Bangladesh. IIED Working Paper. IIED, London. <http://pubs.iied.org/16605IIED/?a=K+Hasan> (accessed: October 27^th^ 2017).

Islam, M., Mohammed, E.Y., Ali, L., 2016. Economic incentives for sustainable hilsa fishing in Bangladesh: An analysis of the legal and institutional framework. Mar. Policy 68, 8–22.

Miah, M.S., 2015. Climatic and anthropogenic factors changing spawning pattern and production zone of Hilsa fishery in the Bay of Bengal. Weather Clim. Extrem. 1–7. doi:10.1016/j.wace.2015.01.001

Milton, D.A., Chenery, S.R., 2001. Can otolith chemistry detect the population structure of the shad hilsa *Tenualosa ilisha*? Comparison with the results of genetic and morphological studies. Mar. Ecol. Prog. Ser. 222, 239–251.

Mome, M.A., Arnason, R., 2007. The potential of the artisanal hilsa fishery in Bangladesh: an economically efficient fisheries policy. Final Project, The United Nations University. <http://www.unuftp.is/static/fellows/document/masud07prf.pdf> (accessed: October 27^th^ 2017).

Rahman, M.J., Cowx, I.G., 2008. Population dynamics of hilsa shad (*Tenualosa ilisha*, Clupeidae) in Bangladesh waters. Asian Fish. Sci. 21, 85–100.

Rahman, Alam, M.A., Hasan, S.J., Zaher, M., 2012a. Biology and ecology of hilsa shad, *Tenualosa ilisha* (Ham.) In: Anon (Ed.) Hilsa: Status of fishery and potential for aquaculture, proceedings of the regional workshop held in Dhaka, 16-17 September 2012. The WorldFish Bangladesh and South Asia Office, Dhaka, pp. 1-39.

Rahman, Alam, M.A., Hasan, S.J., Zaher, M., 2012b. Hilsa (*Tenualosa ilisha*) fishery management in Bangladesh. In: Anon (Ed.) Hilsa: Status of fishery and potential for aquaculture, proceedings of the regional workshop held in Dhaka, 16-17 September 2012. The WorldFish Bangladesh and South Asia Office, Dhaka, pp. 40-60.

Salini, J., Milton, D., Rahman, M., Hussain, M., 2004. Allozyme and morphological variation throughout the geographic range of the tropical shad, hilsa *Tenualosa ilisha*. Fish. Res. 66, 53–69. doi:10.1016/S0165-7836(03)00124-3

Sharma, R., 2012. Report of the hilsa fisheries assessment working group II, 24-25 April 2012, Mumbai, India. BOBLME-2012-HFAWG. Bay of Bengal Large Marine Ecosystem Project, Mumbai, India.
